# STING‐ATF3/type I interferon crosstalk: A potential target to improve anti‐tumour immunity in chemotherapy‐treated urothelial carcinoma

**DOI:** 10.1002/ctm2.70011

**Published:** 2024-09-13

**Authors:** Alexandra Fauvre, Margot Machu, Audrey Merienne, Nadia Vie, Thomas Bessede, Mathilde Robin, Veronique Garambois, Clara Taffoni, Nadine Laguette, Nadine Gervois‐Segain, Anne Jarry, Nathalie Labarriere, Yves Allory, Christel Larbouret, Laurent Gros, Diego Tosi, David B. Solit, Philippe Pourquier, Nadine Houédé, Celine Gongora

**Affiliations:** ^1^ IRCM Univ Montpellier, Inserm, ICM, CNRS Montpellier France; ^2^ Nantes Université, Univ Angers, INSERM, CNRS Nantes France; ^3^ IGMM, Université de Montpellier, CNRS Montpellier France; ^4^ Institut Curie Paris France; ^5^ Weill Cornell Med Coll New York New York USA

Dear Editor,

In this study, we present the first demonstration that activation of the cGAS‐STING pathway in tumour cells by chemotherapies does not necessarily lead to the production of type I interferon. Indeed, we show that the transcription factor ATF3, also induced by chemotherapies, acts as a transcriptional inhibitor of type I Interferon.

Upper tract urothelial carcinomas (UTUCs) are extremely aggressive and immunosuppressed tumours.^1^ UTUC management is based on the combination of cisplatin and gemcitabine (CisGem) or carboplatin and gemcitabine (CarboGem); however, the relapse rate is > 50%.^2^ Here, we investigated CisGem and CarboGem effects in UTUC (UM‐UC‐14, UCC‐03, UCC‐14 and UCC‐17) and bladder cancer cell lines (HT‐1197 and MB49) to identify additional targets that might improve their efficiency.

First, using a full‐range dose matrix approach^3^ SRB cytotoxicity assays we found that CisGem and CarboGem displayed an additive effect in 2D cultures and areas of synergistic effects in 3D cultures of UM‐UC‐14, HT‐1197, MB49, UCC‐03 and UCC‐17 cells (Figure S1A), independently of their sensitivity (IC_50_ in Figure S1B) to these drugs. Moreover, H2AX, ATM, ATR, CHK1 and CHK2 (but not DNA‐PKcs) phosphorylation was increased in cells incubated with CisGem or CarboGem, indicating DNA damage induction and DNA damage response pathway activation (multiplexed immunofluorescence analysis; Figure S2A).

RNA‐sequencing analysis of UM‐UC‐14 cells incubated with CisGem for 24 h identified 482 upregulated genes (particularly *ATF3*) and 376 downregulated genes (Figure S3A). Gene Set Enrichment Analysis indicated that four of the ten most differentially expressed gene sets were related to inflammation (Figure 1A). The enrichment scores for these four gene sets were high (*p* = 0.0026) and many IFN‐stimulated genes (ISGs) were upregulated (Figure 1B and Figure S3B). We obtained similar results with CarboGem (Figure S3C–E). Moreover, in vitro analysis of calreticulin exposure, ATP release and HMGB1 release (Figure 2C–E) showed that the combinations induce immunogenic cell death markers, unlike cisplatin and carboplatin alone. Both combinations also upregulated *PD‐L1* transcript, protein levels and PD‐L1 cell surface expression (Figure S4A–D).

**FIGURE 1:**
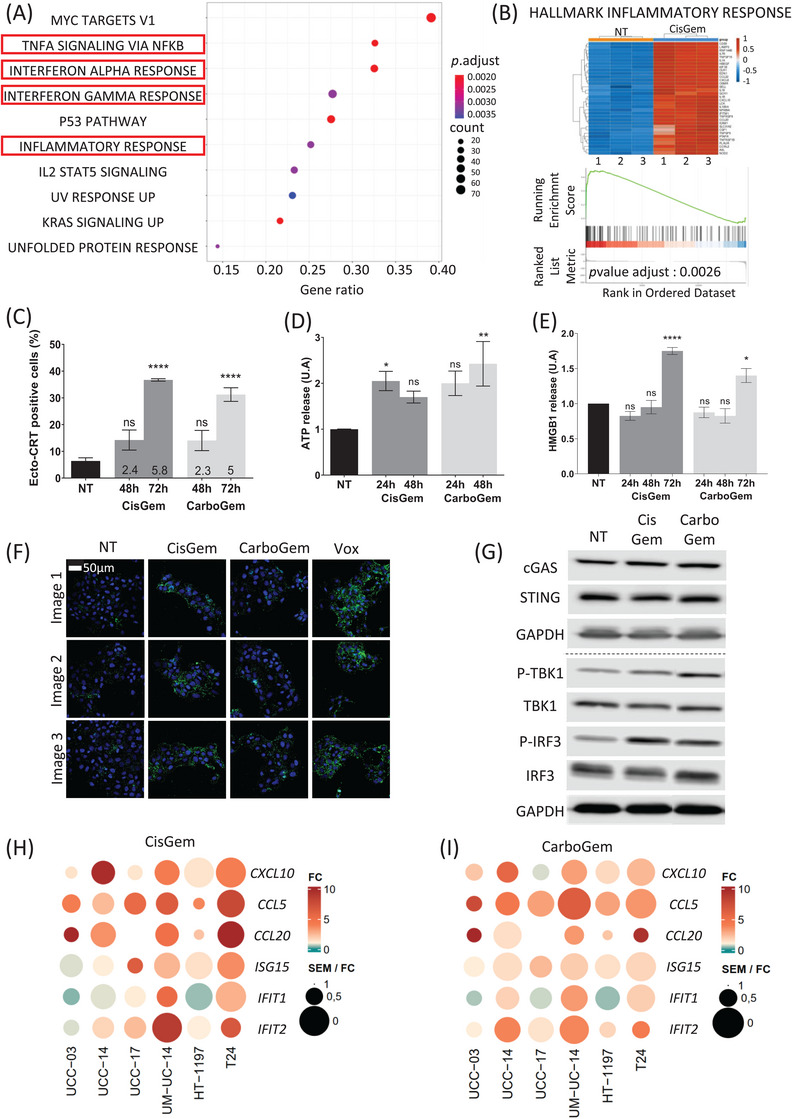
Cisplatin and gemcitabine (CisGem) and carboplatin and gemcitabine (CarboGem) induce inflammatory response pathways. (A) GSEA analysis; NT vs CisGem condition. The first 10 gene sets enriched in the treated condition and ranked according to their gene ratio are shown. Pathways involved in inflammation are framed in red. (B) Heatmap showing the core genes (CPM, count per million) and enrichment plots for the “hallmark inflammatory response” gene set (NT vs CisGem). (C‐E) Immune cell death markers: CRT exposure at the cell surface (C) and ATP (D) and HMGB1 (E) release in the supernatants of UM‐UC‐14 cells assessed by flow cytometry after 48‐ or 72‐hour incubation with cisplatin (3.5μM) + gemcitabine (2nM) or carboplatin (29.5μM) + gemcitabine (2nM). Data are the mean ± SEM of at least three independent experiments. (F) Immunofluorescence analysis of UM‐UC‐14 cells incubated or not (NT) with cisplatin (7μM) + gemcitabine (4nM), carboplatin (59μM) + gemcitabine (4nM), or VE‐822 (ATR inhibitor; 1μM) + oxaliplatin (12.5μM) (Vox) as positive control for 24 hours and stained for cytosolic dsDNA (green) and Hoechst (blue). Images were acquired with an Axioimager M2 microscope with Apotome 2 (Zeiss). Images are representative of three independent experiments. (G) Western blot analysis of the cGAS‐STING signaling pathway in UM‐UC‐14 cells incubated or not (NT) with cisplatin (7μM) + gemcitabine (4nM) or carboplatin (59M) + gemcitabine (4nM) for 24 hours. GAPDH was used as loading control. (H‐I) Heatmaps of the SEM/fold change ratio of six ISGs detected by RT‐qPCR in UTUC (UM‐UC‐14) and bladder (HT‐1197 and T24) cancer cell lines and patient‐derived UTUC cells (UCC‐03, UCC‐14 and UCC‐17) incubated with CisGem (H) or CarboGem (I). Heatmaps were obtained with the R software. Color is determined by the mean fold change of treated conditions versus non‐treated condition, of several independent experiments. The circle size is inversely proportional to the SEM.

**FIGURE 2:**
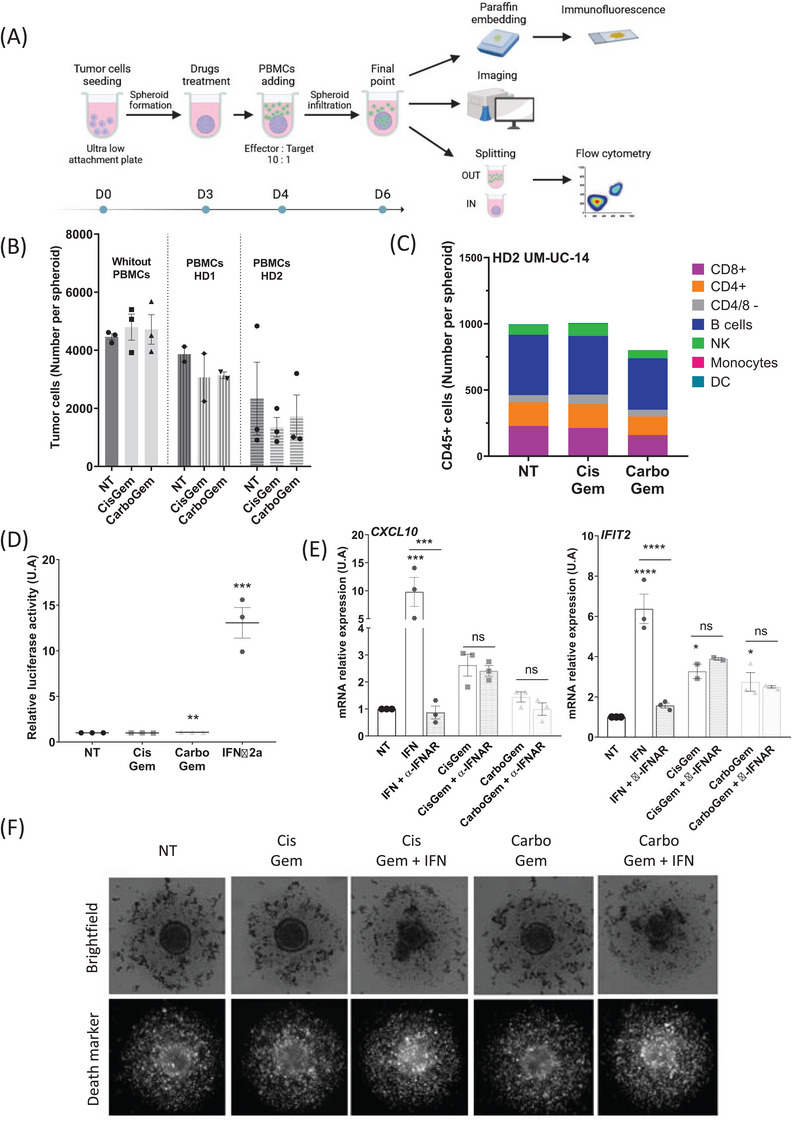
Absence of IFN‐I production after STING pathway activation prevents optimal cisplatin and gemcitabine (CisGem) and carboplatin and gemcitabine (CarboGem) immune system activation. (A) Schematic representation of the co‐culture experimental design. (B) Number of living UM‐UC‐14 cells per spheroid determined by flow cytometry, after co‐culture without or with PBMCs from Healthy Donor #1 (HD1) or #2 (HD2) and incubation or not (NT) with cisplatin (0.125μM) + gemcitabine (0.625nM) or carboplatin (0.625μM) + gemcitabine (0.625nM) for 3 days. (C) Immunophenotyping of spheroid‐infiltrating immune cells (PBMCs from HD2) after incubation with cisplatin (0.125μM) + gemcitabine (0.625nM) or carboplatin (0.625μM) + gemcitabine (0.625nM) for 2 days. NK, natural killer; DC, dendritic cells. (D) Luciferase assay in IFN‐α‐reporter‐expressing HL‐116 cells incubated with conditioned medium from UM‐UC‐14 cells incubated with cisplatin (7μM) + gemcitabine (4nM) or carboplatin (59μM) + gemcitabine (4nM) for 24 hours. IFN‐α‐2a (1000 U/mL) was used as positive control. Data are the mean ± SEM of at least 3 independent experiments. (E) Analysis of CXCL10 and IFIT2 relative expression in UM‐UC‐14 cells by RT‐qPCR after 24‐ hour incubation with IFN‐α‐2a (1000 U/mL), cisplatin (7μM) + gemcitabine (4nM), or carboplatin (59μM) + gemcitabine (4nM) and with/without the anti‐IFNAR antibody (5μg/mL). Data are the mean ± SEM of at least 3 independent experiments. (F) Celigo images of UM‐UC‐14 spheroids co‐cultured with PBMCs (from HD2) and incubated or not (NT) with cisplatin (0.125μM) + gemcitabine (0.625nM) or carboplatin (0.625μM) + gemcitabine (0.625nM), with/without IFN‐α‐2a (1000 U/mL) for 2 days. ns: non‐significant, *P〈0.05, **P〈0.01, ***P〈0.001, ****P〈0.0001 compared with NT.

As DNA damage can activate the cGAS‐STING pathway,^4^, ^5^ particularly by releasing damaged DNA into the cytosol, we monitored the presence of DNA in the cytoplasm, TBK1 (STING target) and IRF3 phosphorylation, and ISG expression (RT‐qPCR) after incubation (or not) with CisGem and CarboGem. The amount of cytosolic DNA was slightly higher (Figure 1F and Figure S2B), and phosphorylation of TBK1 and IRF3 (Figure 1G and Figure S2C), but not of NF‐κB (Figure S4E) was increased in treated than untreated cultures. Moreover, CisGem and CarboGem induced expression of ISGs (Figure 1H,I and Figure S2E), but not *IFNB*. ISG upregulation was stronger upon incubation with IFN‐α−2a than with CisGem or CarboGem alone (Figure S2D). These results indicate that the CisGem and CarboGem activate the cGAS‐STING pathway, but not optimally because IFN‐I induction was undetectable and ISG induction was weak.

To determine the molecular mechanisms involved in cGAS‐STING pathway activation by CisGem and CarboGem, we knocked out cGAS or STING in UM‐UC‐14 cells using the CRISPR/Cas9 technology. Upon incubation with CisGem and CarboGem, TBK1 and IRF3 phosphorylation was increased (Figure S5B‐C) and *CXCL10* and *IFIT1* (two ISGs) were upregulated in UM‐UC‐14^cGAS ‐/−^ but not in UM‐UC‐14^STING ‐/−^ cells (Figure S5D). Sensitivity to the two combinations (IC_50_) was similar in UM‐UC‐14^cGAS ‐/‐^ and UM‐UC‐14^STING ‐/−^ cells, control and parental cells (Figure S5A). Therefore, drug sensitivity is cGAS‐ and STING‐independent and ISG induction is STING‐dependent in UTUC cell lines.

To understand the role of STING pathway activation by CisGem and CarboGem in anti‐tumour immunity, we generated UM‐UC‐14 cell spheroids and after 3 days we added CisGem or CarboGem (Figure 2A). The next day, we added interleukin‐15‐activated peripheral blood mononuclear cells (PBMCs) from four healthy donors. After 2, 3, or 4 days of co‐culture, we dissociated the spheroids for flow cytometry or immunofluorescence imaging analysis. In non‐treated spheroids, PBMCs had the expected allogeneic effect (Figure 2B), the magnitude of which was donor‐dependent. CisGem and CarboGem did not enhance this effect (Figure 2B). Moreover, PBMCs infiltrated the spheroids (300–1000 PBMCs per UM‐UC‐14 cell spheroid), particularly B cells and CD4^+^ and CD8^+^ T cells (Figure 2C and Figure S6C,D). CisGem and CarboGem neither increased the number (Figure 2C and Figure S8B) nor changed the nature of the infiltrated cells (Figure 2C). PBMC immunophenotyping indicated that immune cell infiltration in spheroids was a specific process and did not rely solely on the proportion of immune cell types in the starting sample (Figure S6B). These results suggest that CisGem and CarboGem did not affect the number and type of spheroid‐infiltrating immune cells (indicating suboptimal inflammation induction) and did not activate the anti‐tumour immunity. This could be explained by defective IFN‐I production. Indeed, upon incubation of cells expressing luciferase under the control of an IFNα‐responsive promoter with conditioned medium from UM‐UC‐14, UCC‐03 or UCC‐17 cells exposed to CisGem or CarboGem, we did not detect any luciferase activity. This indicated the absence of IFN‐α in the conditioned media, and thus no IFN induction upon chemotherapy (Figure 2D and Figure S7A,B). Moreover, a blocking anti‐IFNAR antibody (Figure 2E and Figure S7C) prevented ISG upregulation (*IFIT1, IFIT2, CXCL10* and *CCL20*) by IFN‐I, but not by CisGem or CarboGem, indicating that ISG upregulation by the combinations is IFN‐independent. Lastly, when spheroids (co‐cultured with PBMCs) were incubated with CisGem or CarboGem and IFNα−2a, their size was reduced and cell death increased (Figure 2F). This indicates that IFN‐I is essential for the immune cell cytotoxic effects on tumour cells.

The transcription factor *ATF3* was one of the genes and proteins most upregulated by CisGem and CarboGem (Figures S3A,C and S7D and Figure 3A). As ATF3 prevents IFN‐I induction upon viral infection of monocytes,^6^ we asked whether ATF3 induction might explain the lack of IFN‐I production upon incubation with CisGem or CarboGem. In HT‐29*^ATF3‐/−^* cells (CRISPR/Cas9‐based deletion), IFN‐I was upregulated upon incubation with CisGem and CarboGem (Figure 3B,C). Moreover, ISG upregulation by CisGem and CarboGem was higher in HT‐29*^ATF3‐/−^* than parental cells (Figure 3D and Figure S7E). Therefore, in cultured cancer cells, CisGem and CarboGem activate the STING pathway in a non‐optimal manner because ATF3 inhibits IFN‐I production. Moreover, HT29^ATF3‐/−^ spheroids (co‐cultured with PBMCs) lost their integrity upon PBMC addition compared with HT29^CTL^ spheroids, and displayed increased cell death (Figure S8A), indicating a better anti‐tumour effect of PBMCs. In agreement, compared with HT29^CTL^ spheroids, the number of tumour cells was decreased and that of infiltrating PBMCs increased in HT29^ATF3 ‐/−^ spheroids (Figure 3E,F and Figure S8E). The increase in B and CD8^+^ T cells (numbers and percentages) in HT29^ATF3‐/‐^ spheroids was donor‐independent (immunophenotyping in Figure 3G,H and Figure S8C,D). Thus, *ATF3* KO enhances anti‐tumour immunity and favours immune cell infiltration.

**FIGURE 3:**
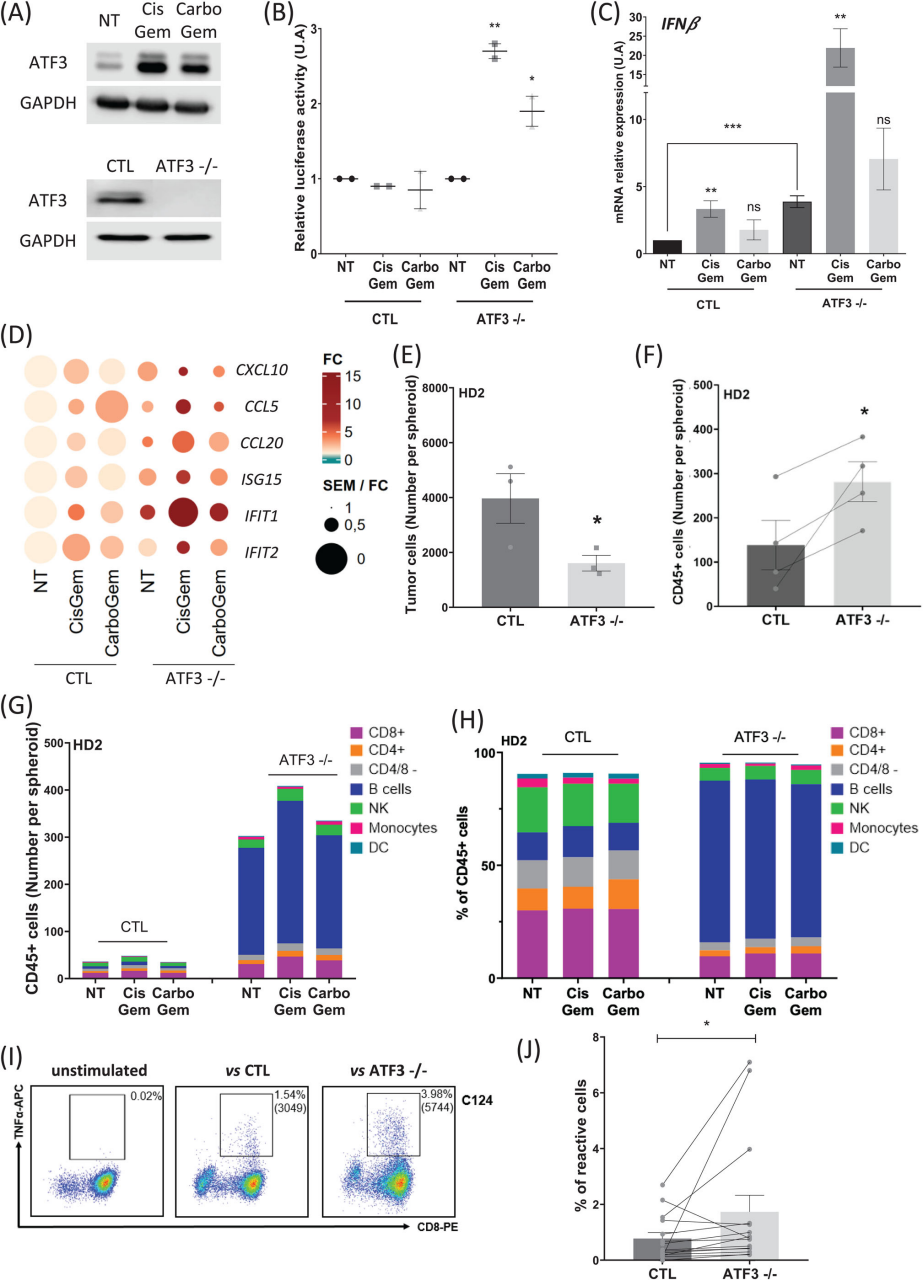
ATF3 is induced by chemotherapy and ATF3 KO increases immune cell infiltration and activation leading to cancer cell death. A. Western blot analysis of ATF3 expression in UM‐UC‐14 cells incubated or not (NT) with cisplatin (7μM) + gemcitabine (4nM) or carboplatin (59μM) + gemcitabine (4nM) for 24 hours, and in HT‐29CTL and HT‐29ATF3‐/‐ cells. GAPDH was used as loading control. B. Luciferase assay in IFN‐α‐reporter‐expressing HL‐116 cells incubated with conditioned medium from control or ATF3‐/‐ cells incubated with cisplatin (7μM) + gemcitabine (4nM) or carboplatin (59μM) + gemcitabine (4nM) for 24 hours. Data are the mean ± SEM of at least three independent experiments. C. Analysis of IFNβ relative expression in HT‐29CTL and HT‐29ATF3‐/‐ cells by RT‐qPCR after 24‐hour incubation with cisplatin (7μM) + gemcitabine (4nM) or carboplatin (59μM) + gemcitabine (4nM). D. Heatmaps of the SEM/fold change ratio of all ISGs detected by RT‐qPCR in HT‐29CTL and HT‐29ATF3‐/‐ cells incubated with CisGem or CarboGem. Heatmaps were obtained with the R software. Color is determined by the mean fold change of treated conditions versus non‐treated condition (several independent experiments). The circle size is inversely proportional to the SEM. E. Number of living tumor cells per spheroid in HT‐29CTL and HT‐29ATF3‐/‐ spheroids co‐cultured with HD2 PBMCs. F. Number of living CD45+ cells per spheroid in HT‐29CTL and HT‐29ATF3‐/‐ spheroids co‐cultured with HD2 PBMCs. G‐H. Immunophenotyping of the immune cells (HD2 PBMCs) that infiltrated HT‐29CTL and HT‐29ATF3‐/‐ spheroids, numbers (G) and percentages (H), incubated with cisplatin (0.175μM) + gemcitabine (0.112nM) or carboplatin (1.6μM) + gemcitabine (0.112nM) for 3 days. NK, natural killer cells; DC, dendritic cells. I. Density plots showing the TNF‐α response in CRC‐derived CD8+ TIL populations (sample C124) when cultured alone (left) and after co‐culture with HT‐29CTL (middle) or with HT‐29ATF3‐/‐ cells (right). Results are expressed as % of TNF‐α‐producing cells; MFI values are between brackets. J. Percentage of TNF‐α‐producing CD8+ TILs after co‐culture with HT‐29CTL and HT‐29ATF3‐/‐ cells (*n* = 15). ns: not significant, *P〈0.05, **P〈0.01, ***P〈0.001, ****P〈0.0001 compared with untreated condition (NT).

Lastly, we analyzed whether HT‐29^ATF3‐/−^ and HT‐29^CTL^ cells stimulate CD8^+^ tumour‐infiltrating lymphocytes (TILs) (*n* = 15 samples from patients with colorectal cancer) by measuring TNF‐α production (Figure 3I). TNF‐α production was increased in 13/15 TIL populations co‐cultured with HT‐29^ATF3‐/‐^ cells compared with HT‐29^CTL^ cells, and the mean fluorescence intensity was increased in 7/15 TIL samples (Figure S8F and Figure 3I shows data for CD8^+^ TILs from patient C124), The TNF‐α production increase was significant in percentage (*p *= 0.0466) and MFI (*p *= 0.0418) (Figure 3J and Figure S8F). This again indicates that ATF3 expression/activity inhibits the anti‐tumour immune response, particularly by inhibiting CD8^+^ T‐cell activation.

This study showed that CisGem and CarboGem activation of the STING pathway is suboptimal due to IFN‐I production inhibition by ATF3 upregulation. As the absence of IFN‐I production in UTUC cells might negatively affect the anti‐tumour immune response, CisGem and CarboGem could be combined with ATF3 inhibitors or with IFN‐I.

## AUTHOR CONTRIBUTIONS

*Conceptualization*: Nadine Houédé, CG, Alexandra Fauvre, Margot Machu, Philippe Pourquier. *Methodology*: Alexandra Fauvre, Margot Machu, Audrey Merienne, Nadia Vie, Thomas Bessede, Mathilde Robin, Veronique Garambois, CM, and Clara Taffoni. *Investigation*: Alexandra Fauvre, Margot Machu, Audrey Merienne, Nadia Vie, Thomas Bessede, Mathilde Robin, Veronique Garambois, CM, Clara Taffoni, Nadine Houédé, CG, Christel Larbouret, Diego Tosi, and Laurent Gros. *Funding acquisition*: Nadine Houédé, CG, Philippe Pourquier. *Project administration*: Nadine Houédé, CG, Christel Larbouret, Diego Tosi, Yves Allory, David B. Solit. *Supervision*: Nadine Houédé, CG, Philippe Pourquier. *Writing—original draft*: Nadine Houédé, CG, Alexandra Fauvre, Margot Machu. *Writing—review and editing*: Nadine Houédé, CG, Alexandra Fauvre, Margot Machu, Philippe Pourquier, Laurent Gros, Nadine Laguette, Nathalie Labarriere, Nadine Gervois‐Segain, and Anne Jarry.

## FUNDING INFORMATION

This study is supported bu the SIRICMontpellier Cancer Grant INCa_Inserm_DGOS_12553, REACT‐EU (Recovery Assistancefor Cohesion and the Territories of Europe), GIS FC3R (funds managed by Inserm, IBiSA, Ligue Contre le Cancer and Occitanie Region), French National Research Agency (ANR‐10‐INBS‐04, Investments for the Future), Grant Agreement LabExMAbImprove: ANR‐10‐LABX‐53, Investments for the Future.

## ETHICS STATEMENT

Allhuman studies were reviewed and approved by the appropriate institutional review board/ethics committee and were performed in accordance with the ethical standards of the authors.

## Supporting information

Supporting information
