# Supplementary material for: STING‐ATF3/type I interferon crosstalk: A potential target to improve anti‐tumour immunity in chemotherapy‐treated urothelial carcinoma
Source: Clin Transl Med. 2024 Sep 13;14(9):e70011. doi: 10.1002/ctm2.70011 (PMC11397130; doi:10.1002/ctm2.70011)
Supplement: Supplementary file 1 — Supporting information [file CTM2-14-e70011-s001.pptx]

## Slide 1
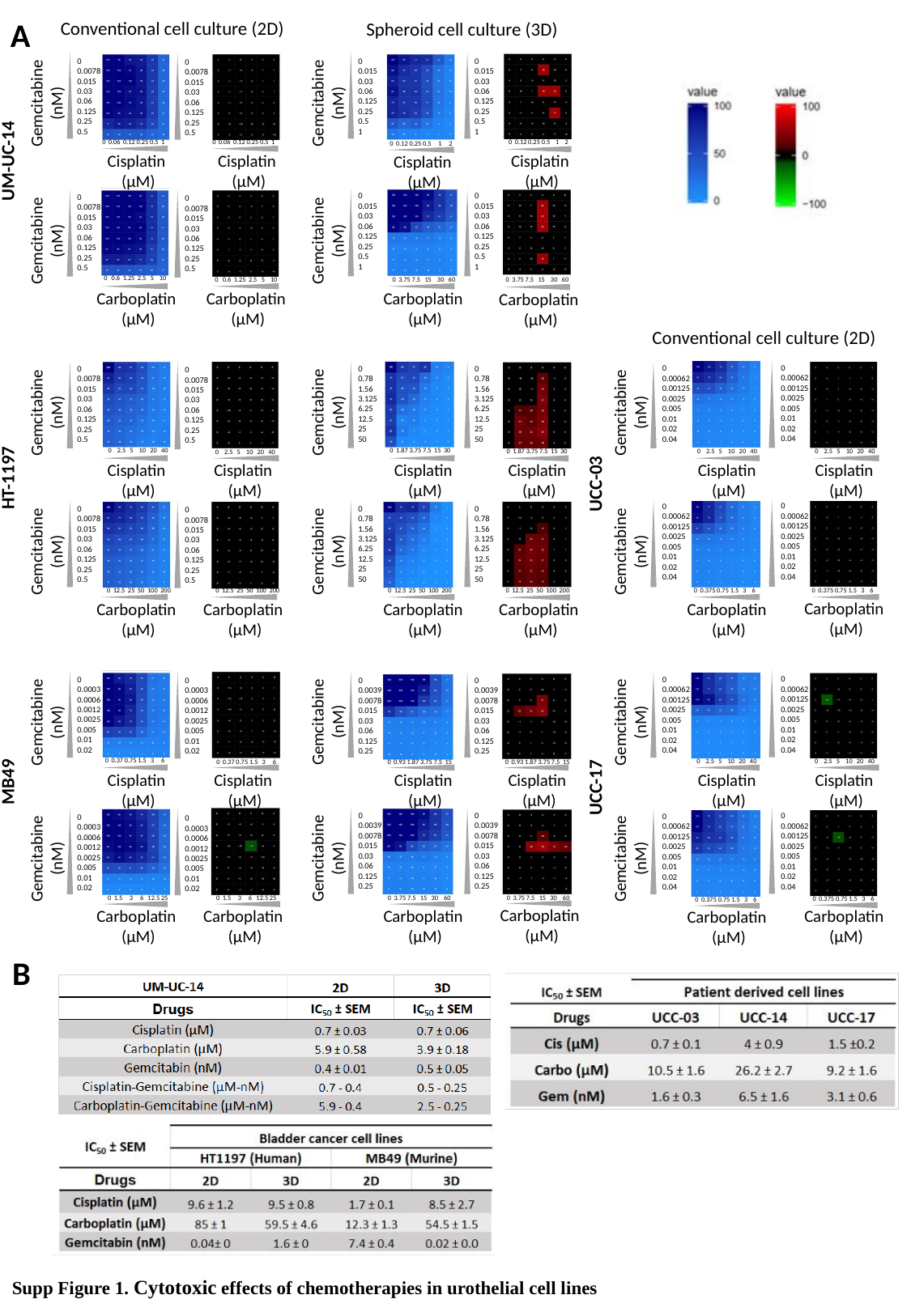

A
Conventional cell culture (2D)
Spheroid cell culture (3D)
0
0.0078
0.015
0.03
0.06
0.125
0.25
0.5
0
0.015
0.03
0.06
0.125
0.25
0.5
1
0
0.015
0.03
0.06
0.125
0.25
0.5
1
0
0.0078
0.015
0.03
0.06
0.125
0.25
0.5
Gemcitabine (nM)
Gemcitabine (nM)
UM-UC-14
0 0.12 0.25 0.5 1 2
0 0.06 0.12 0.25 0.5 1
0 0.06 0.12 0.25 0.5 1
0 0.12 0.25 0.5 1 2
Cisplatin
(µM)
Cisplatin
(µM)
Cisplatin
(µM)
Cisplatin
(µM)
0
0.015
0.03
0.06
0.125
0.25
0.5
1
0
0.015
0.03
0.06
0.125
0.25
0.5
1
0
0.0078
0.015
0.03
0.06
0.125
0.25
0.5
0
0.0078
0.015
0.03
0.06
0.125
0.25
0.5
Gemcitabine (nM)
Gemcitabine (nM)
0 0.6 1.25 2.5 5 10
0 0.6 1.25 2.5 5 10
0 3.75 7.5 15 30 60
0 3.75 7.5 15 30 60
Carboplatin
(µM)
Carboplatin
(µM)
Carboplatin
(µM)
Carboplatin
(µM)
Conventional cell culture (2D)
0
0.00062
0.00125
0.0025
0.005
0.01
0.02
0.04
0
0.00062
0.00125
0.0025
0.005
0.01
0.02
0.04
0
0.78
1.56
3.125
6.25
12.5
25
50
0
0.78
1.56
3.125
6.25
12.5
25
50
0
0.0078
0.015
0.03
0.06
0.125
0.25
0.5
0
0.0078
0.015
0.03
0.06
0.125
0.25
0.5
Gemcitabine (nM)
Gemcitabine (nM)
Gemcitabine (nM)
HT-1197
UCC-03
0 2.5 5 10 20 40
0 1.87 3.75 7.5 15 30
0 2.5 5 10 20 40
0 1.87 3.75 7.5 15 30
0 2.5 5 10 20 40
0 2.5 5 10 20 40
0 2.5 5 10 20 40
Cisplatin
(µM)
Cisplatin
(µM)
Cisplatin
(µM)
Cisplatin
(µM)
Cisplatin
(µM)
Cisplatin
(µM)
0
0.00062
0.00125
0.0025
0.005
0.01
0.02
0.04
0
0.00062
0.00125
0.0025
0.005
0.01
0.02
0.04
0
0.78
1.56
3.125
6.25
12.5
25
50
0
0.78
1.56
3.125
6.25
12.5
25
50
0
0.0078
0.015
0.03
0.06
0.125
0.25
0.5
0
0.0078
0.015
0.03
0.06
0.125
0.25
0.5
Gemcitabine (nM)
Gemcitabine (nM)
Gemcitabine (nM)
0 0.375 0.75 1.5 3 6
0 0.375 0.75 1.5 3 6
0 12.5 25 50 100 200
0 12.5 25 50 100 200
0 12.5 25 50 100 200
0 12.5 25 50 100 200
Carboplatin
(µM)
Carboplatin
(µM)
Carboplatin
(µM)
Carboplatin
(µM)
Carboplatin
(µM)
Carboplatin
(µM)
0
0.00062
0.00125
0.0025
0.005
0.01
0.02
0.04
0
0.0003
0.0006
0.0012
0.0025
0.005
0.01
0.02
0
0.00062
0.00125
0.0025
0.005
0.01
0.02
0.04
0
0.0003
0.0006
0.0012
0.0025
0.005
0.01
0.02
0
0.0039
0.0078
0.015
0.03
0.06
0.125
0.25
0
0.0039
0.0078
0.015
0.03
0.06
0.125
0.25
Gemcitabine (nM)
Gemcitabine (nM)
Gemcitabine (nM)
MB49
UCC-17
0 0.37 0.75 1.5 3 6
0 2.5 5 10 20 40
0 2.5 5 10 20 40
0 0.93 1.87 3.75 7.5 15
0 0.37 0.75 1.5 3 6
0 0.93 1.87 3.75 7.5 15
Cisplatin
(µM)
Cisplatin
(µM)
Cisplatin
(µM)
Cisplatin
(µM)
Cisplatin
(µM)
Cisplatin
(µM)
0
0.0039
0.0078
0.015
0.03
0.06
0.125
0.25
0
0.0039
0.0078
0.015
0.03
0.06
0.125
0.25
0
0.00062
0.00125
0.0025
0.005
0.01
0.02
0.04
0
0.00062
0.00125
0.0025
0.005
0.01
0.02
0.04
0
0.0003
0.0006
0.0012
0.0025
0.005
0.01
0.02
0
0.0003
0.0006
0.0012
0.0025
0.005
0.01
0.02
Gemcitabine (nM)
Gemcitabine (nM)
Gemcitabine (nM)
0 1.5 3 6 12.5 25
0 1.5 3 6 12.5 25
0 3.75 7.5 15 30 60
0 3.75 7.5 15 30 60
0 0.375 0.75 1.5 3 6
0 0.375 0.75 1.5 3 6
Carboplatin
(µM)
Carboplatin
(µM)
Carboplatin
(µM)
Carboplatin
(µM)
Carboplatin
(µM)
Carboplatin
(µM)
B
Supp Figure 1. Cytotoxic effects of chemotherapies in urothelial cell lines

## Slide 2
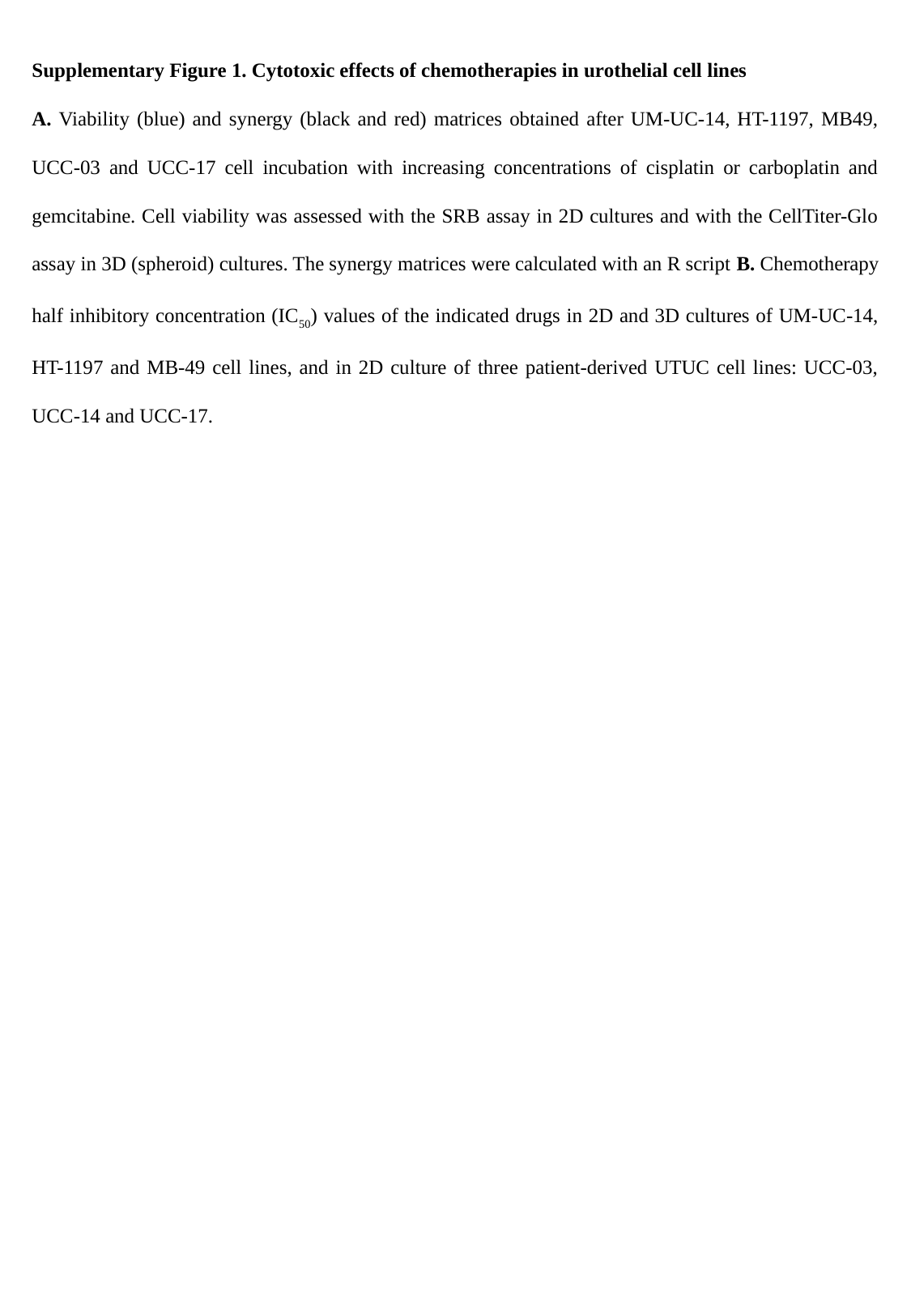

Supplementary Figure 1. Cytotoxic effects of chemotherapies in urothelial cell lines
A. Viability (blue) and synergy (black and red) matrices obtained after UM-UC-14, HT-1197, MB49, UCC-03 and UCC-17 cell incubation with increasing concentrations of cisplatin or carboplatin and gemcitabine. Cell viability was assessed with the SRB assay in 2D cultures and with the CellTiter-Glo assay in 3D (spheroid) cultures. The synergy matrices were calculated with an R script B. Chemotherapy half inhibitory concentration (IC50) values of the indicated drugs in 2D and 3D cultures of UM-UC-14, HT-1197 and MB-49 cell lines, and in 2D culture of three patient-derived UTUC cell lines: UCC-03, UCC-14 and UCC-17.

## Slide 3
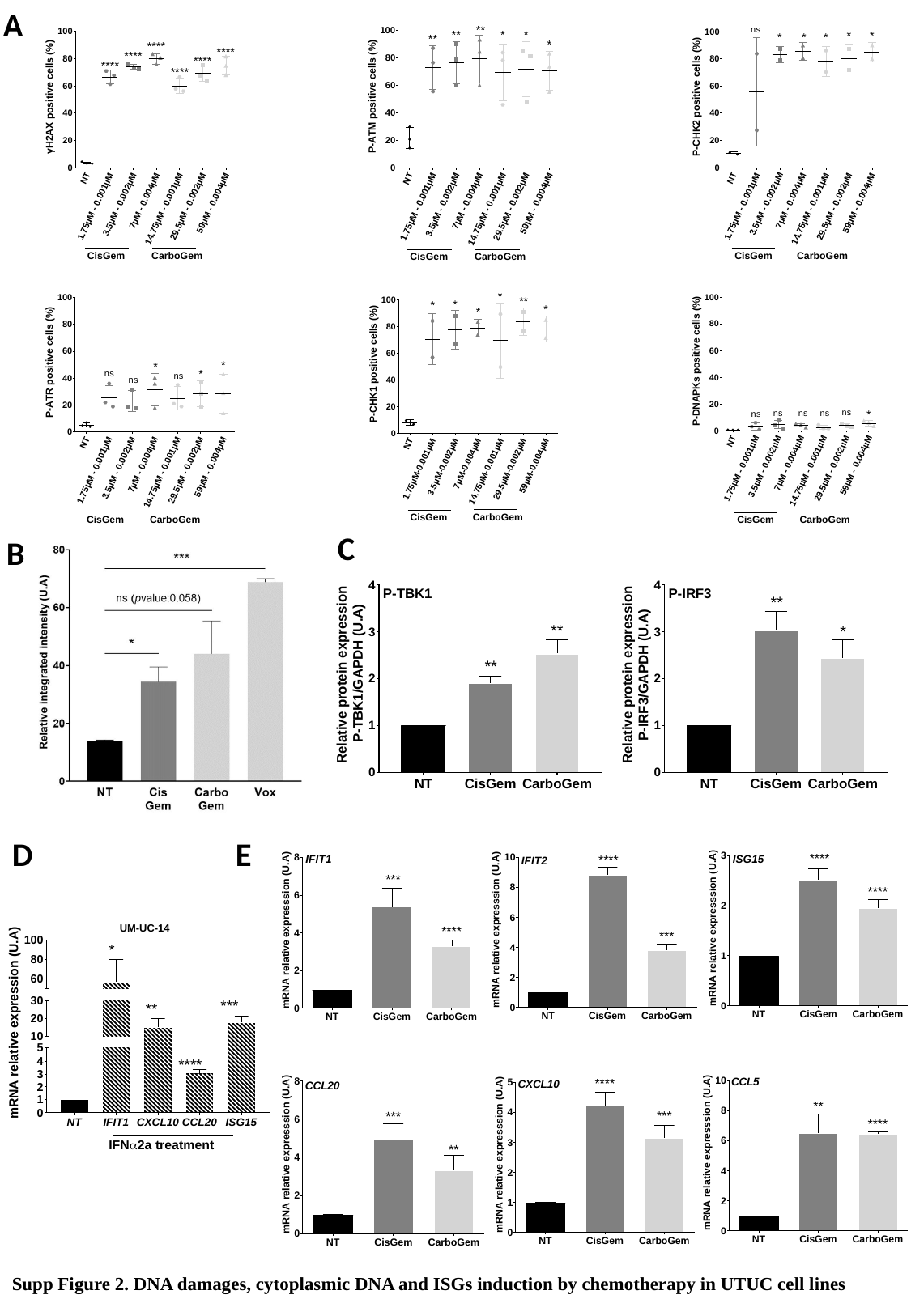

A
C
B
D
E
Supp Figure 2. DNA damages, cytoplasmic DNA and ISGs induction by chemotherapy in UTUC cell lines

## Slide 4
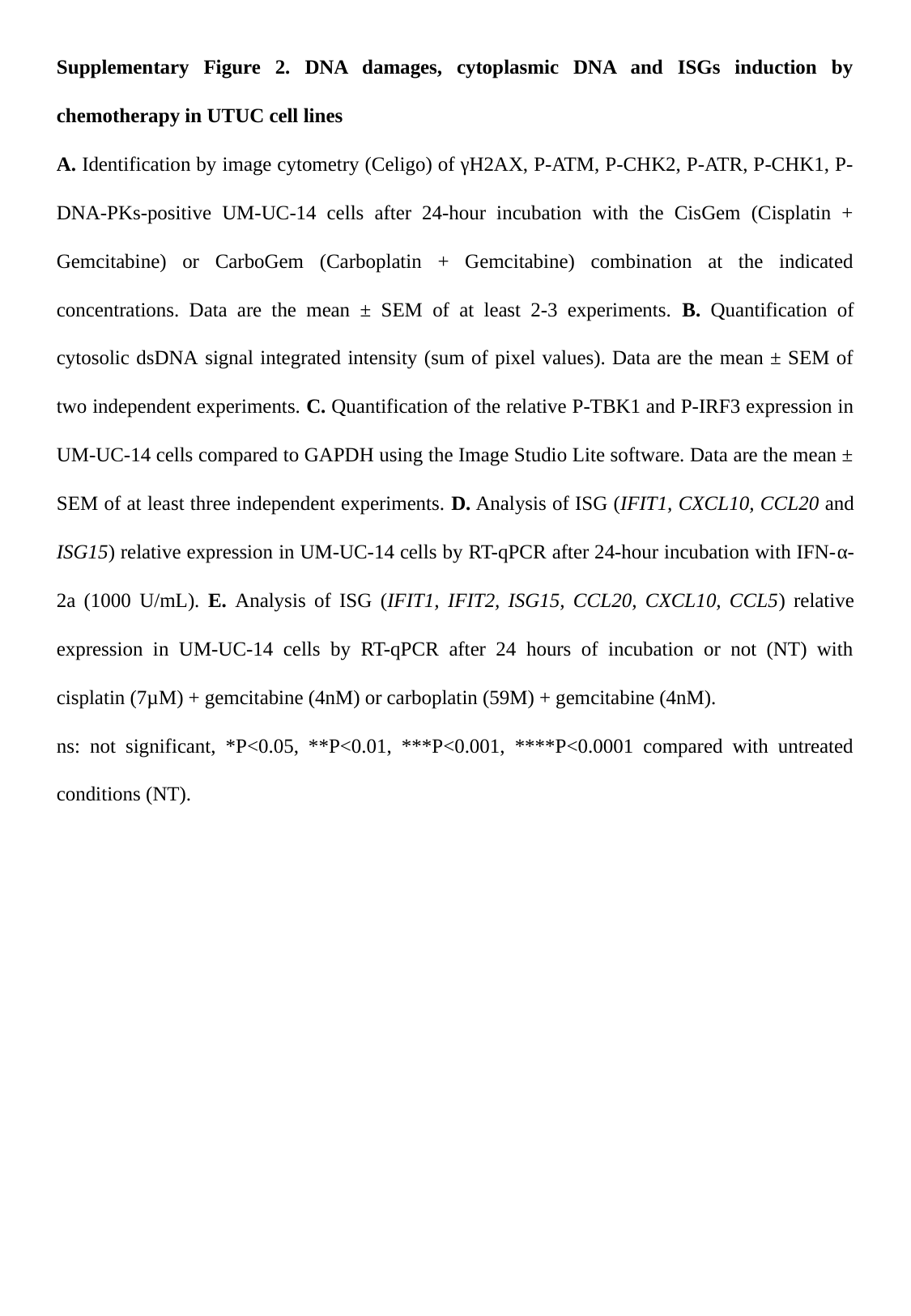

Supplementary Figure 2. DNA damages, cytoplasmic DNA and ISGs induction by chemotherapy in UTUC cell lines
A. Identification by image cytometry (Celigo) of γH2AX, P-ATM, P-CHK2, P-ATR, P-CHK1, P-DNA-PKs-positive UM-UC-14 cells after 24-hour incubation with the CisGem (Cisplatin + Gemcitabine) or CarboGem (Carboplatin + Gemcitabine) combination at the indicated concentrations. Data are the mean ± SEM of at least 2-3 experiments. B. Quantification of cytosolic dsDNA signal integrated intensity (sum of pixel values). Data are the mean ± SEM of two independent experiments. C. Quantification of the relative P-TBK1 and P-IRF3 expression in UM-UC-14 cells compared to GAPDH using the Image Studio Lite software. Data are the mean ± SEM of at least three independent experiments. D. Analysis of ISG (IFIT1, CXCL10, CCL20 and ISG15) relative expression in UM-UC-14 cells by RT-qPCR after 24-hour incubation with IFN-α-2a (1000 U/mL). E. Analysis of ISG (IFIT1, IFIT2, ISG15, CCL20, CXCL10, CCL5) relative expression in UM-UC-14 cells by RT-qPCR after 24 hours of incubation or not (NT) with cisplatin (7µM) + gemcitabine (4nM) or carboplatin (59M) + gemcitabine (4nM).
ns: not significant, *P<0.05, **P<0.01, ***P<0.001, ****P<0.0001 compared with untreated conditions (NT).

## Slide 5
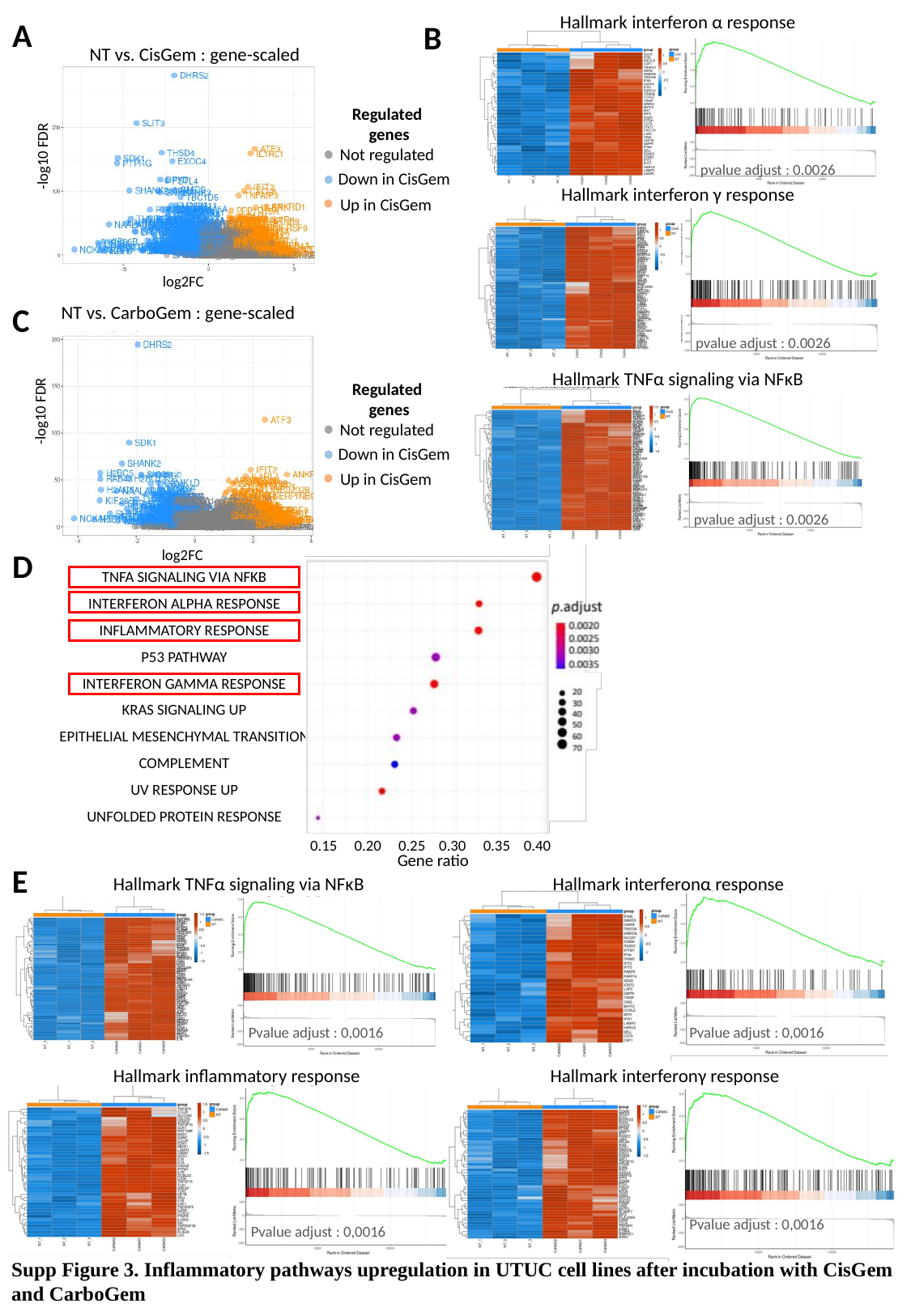

Hallmark interferon α response
A
B
NT vs. CisGem : gene-scaled
-log10 FDR
log2FC
Regulated
genes
Not regulated
Down in CisGem
Up in CisGem
pvalue adjust : 0.0026
Hallmark interferon γ response
pvalue adjust : 0.0026
C
NT vs. CarboGem : gene-scaled
pvalue adjust : 0.0026
Hallmark TNFα signaling via NFκB
Regulated
genes
Not regulated
Down in CisGem
Up in CisGem
-log10 FDR
pvalue adjust : 0.0026
log2FC
D
TNFA SIGNALING VIA NFKB
INTERFERON ALPHA RESPONSE
INFLAMMATORY RESPONSE
P53 PATHWAY
INTERFERON GAMMA RESPONSE
KRAS SIGNALING UP
EPITHELIAL MESENCHYMAL TRANSITION
COMPLEMENT
UV RESPONSE UP
UNFOLDED PROTEIN RESPONSE
0.15 0.20 0.25 0.30 0.35 0.40
Gene ratio
E
Hallmark TNFα signaling via NFκB
Hallmark interferonα response
Pvalue adjust : 0,0016
Pvalue adjust : 0,0016
Hallmark inflammatory response
Hallmark interferonγ response
Pvalue adjust : 0,0016
Pvalue adjust : 0,0016
Supp Figure 3. Inflammatory pathways upregulation in UTUC cell lines after incubation with CisGem and CarboGem

## Slide 6
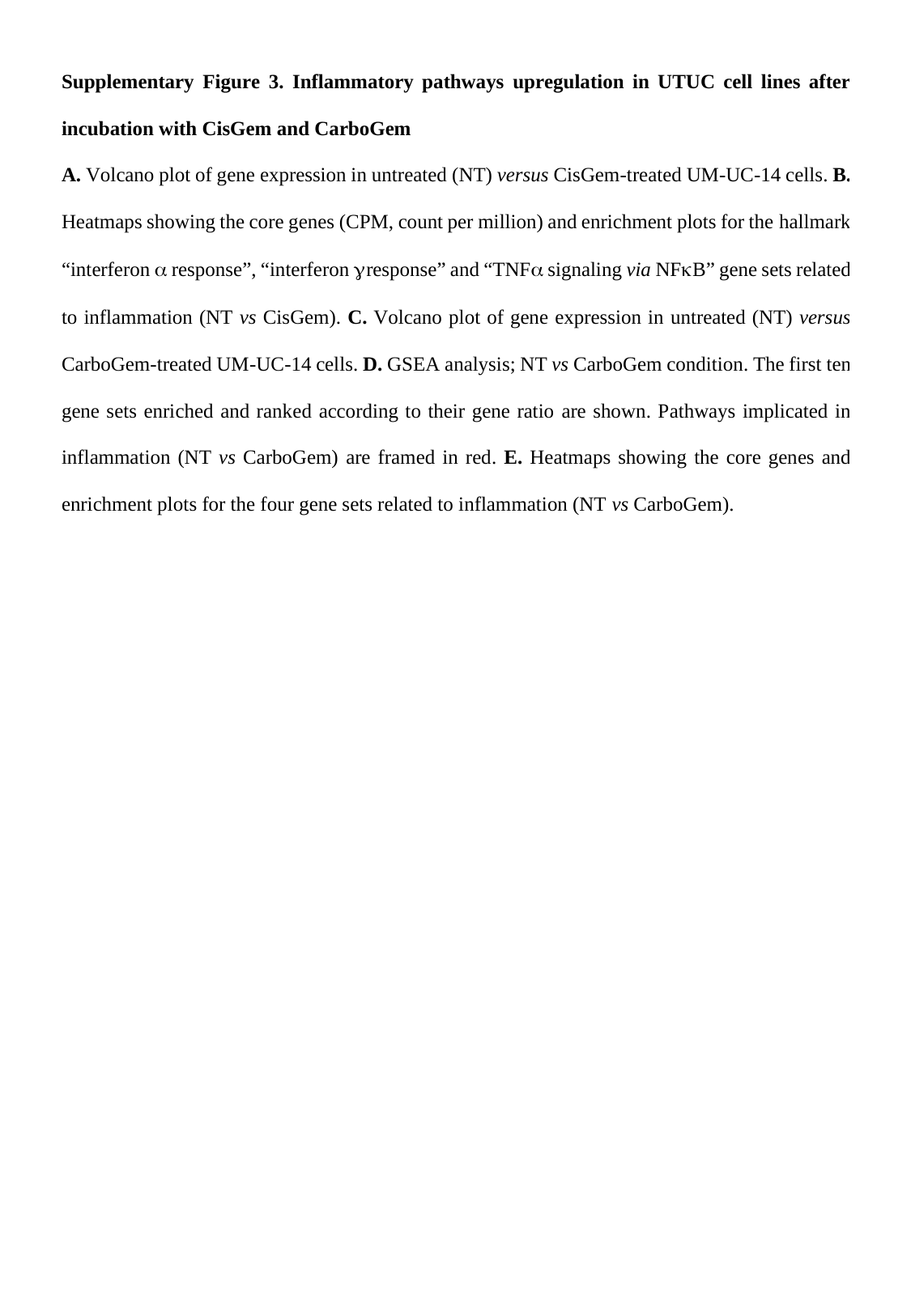

## Slide 7
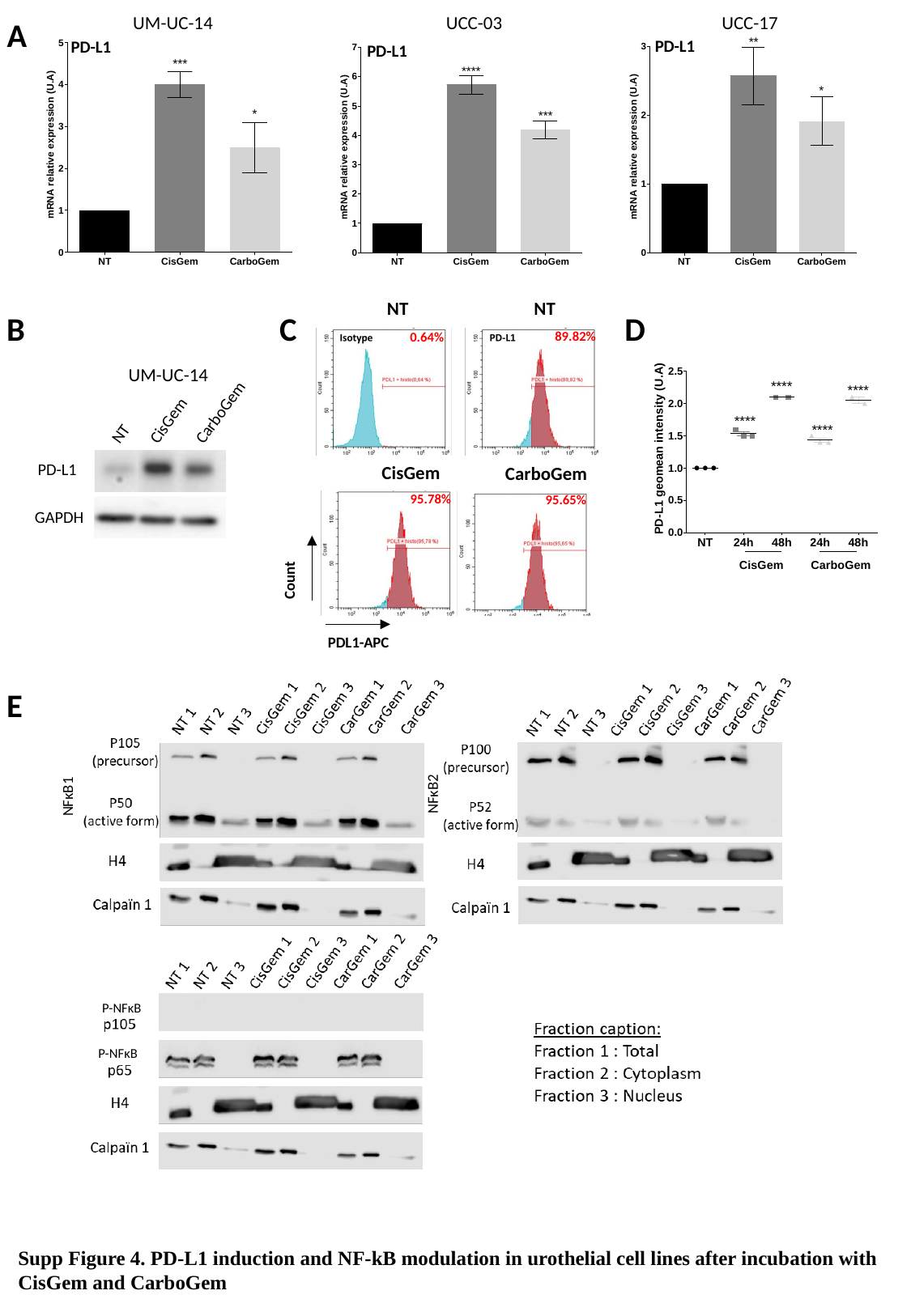

UM-UC-14
UCC-03
UCC-17
A
PD-L1
PD-L1
PD-L1
NT
NT
89.82%
0.64%
CisGem
CarboGem
95.78%
95.65%
PDL1-APC
Count
B
C
D
UM-UC-14
CisGem
CarboGem
NT
PD-L1
GAPDH
NFκB2
NFκB1
P-NFκB
P-NFκB
E
Supp Figure 4. PD-L1 induction and NF-kB modulation in urothelial cell lines after incubation with CisGem and CarboGem

## Slide 8
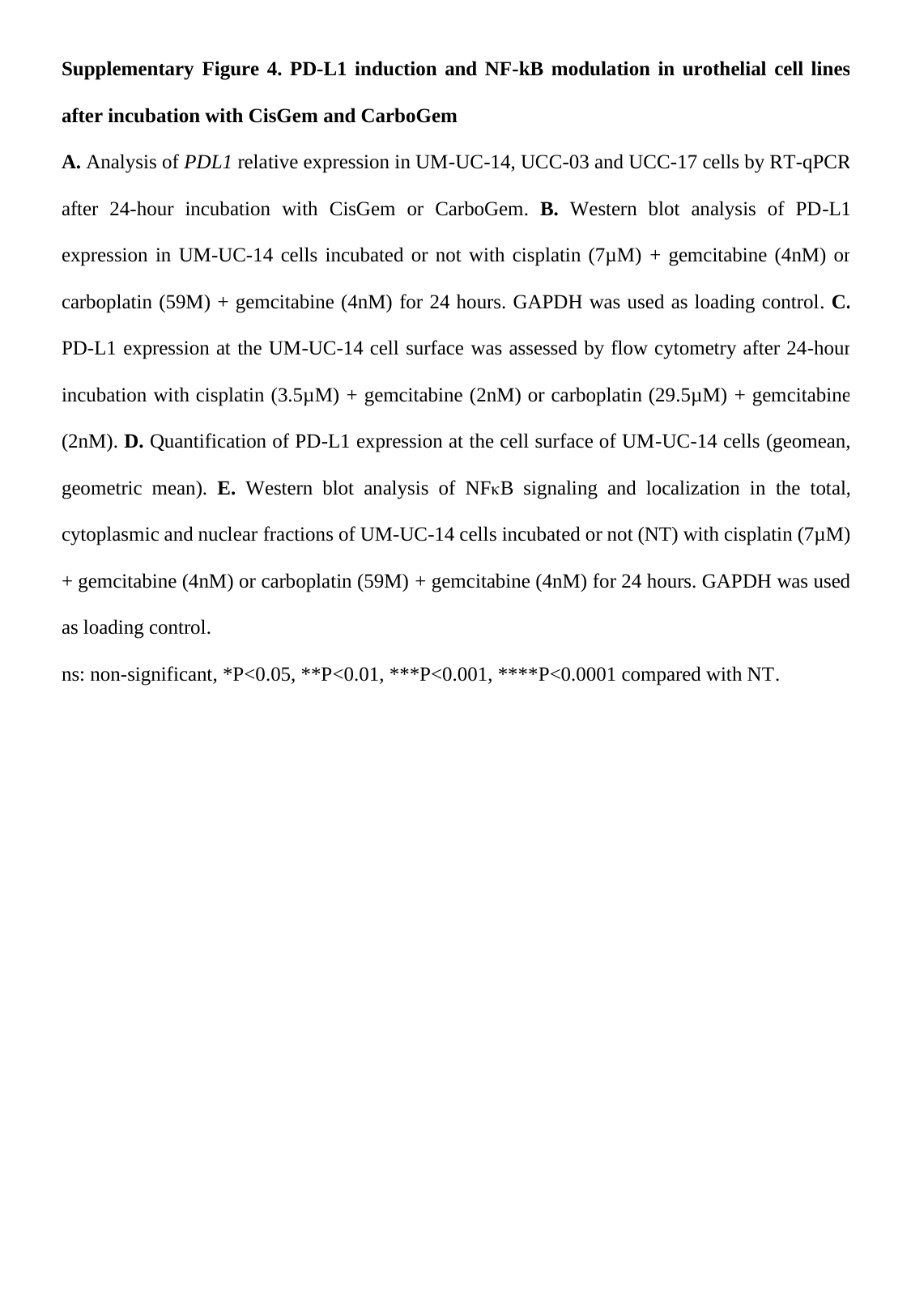

## Slide 9
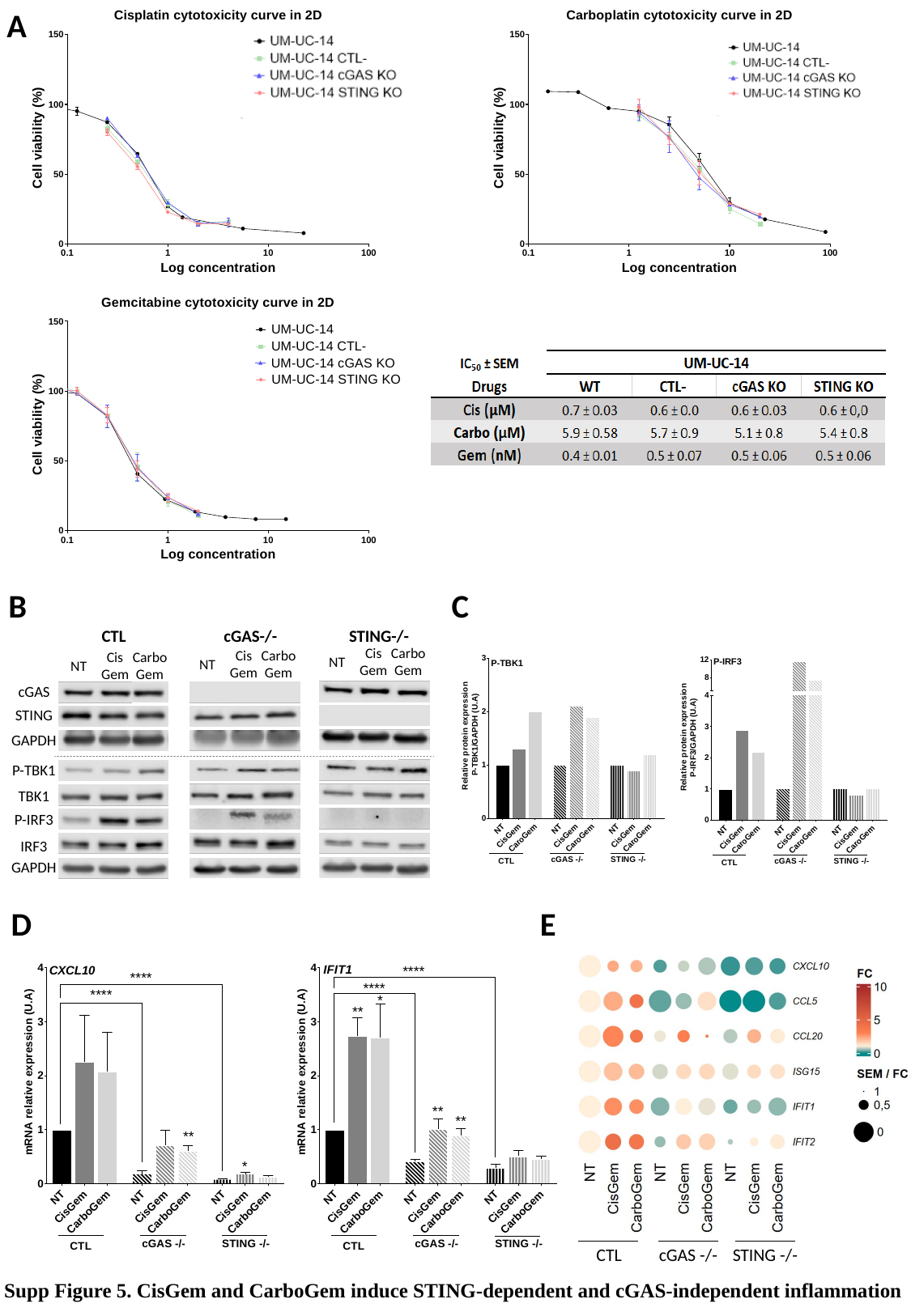

A
B
C
CTL
cGAS-/-
STING-/-
Cis
Gem
Carbo
Gem
NT
Cis
Gem
Carbo
Gem
NT
Cis
Gem
Carbo
Gem
NT
cGAS
STING
GAPDH
P-TBK1
TBK1
P-IRF3
IRF3
GAPDH
D
E
CTL
cGAS -/-
STING -/-
Supp Figure 5. CisGem and CarboGem induce STING-dependent and cGAS-independent inflammation

## Slide 10
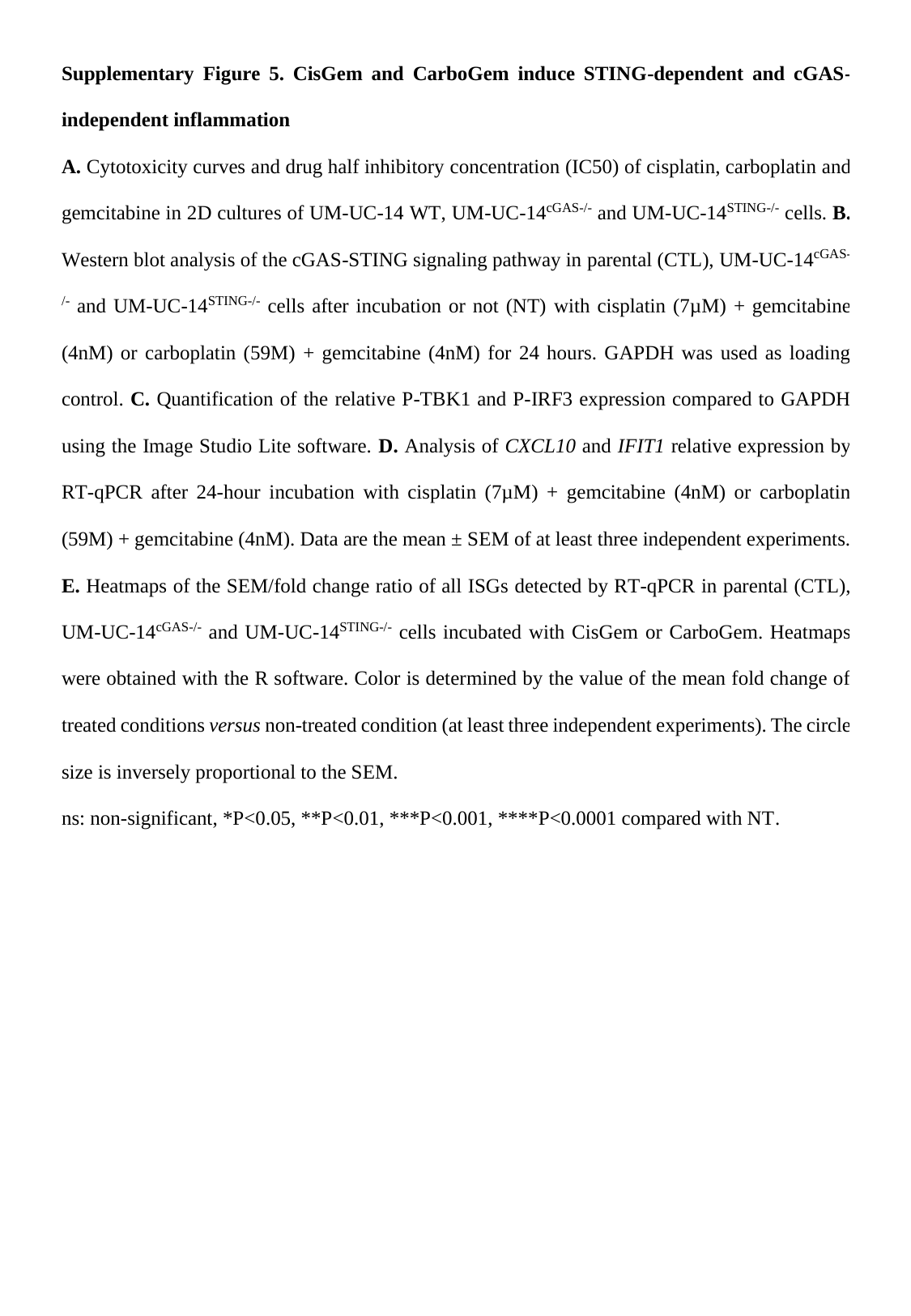

## Slide 11
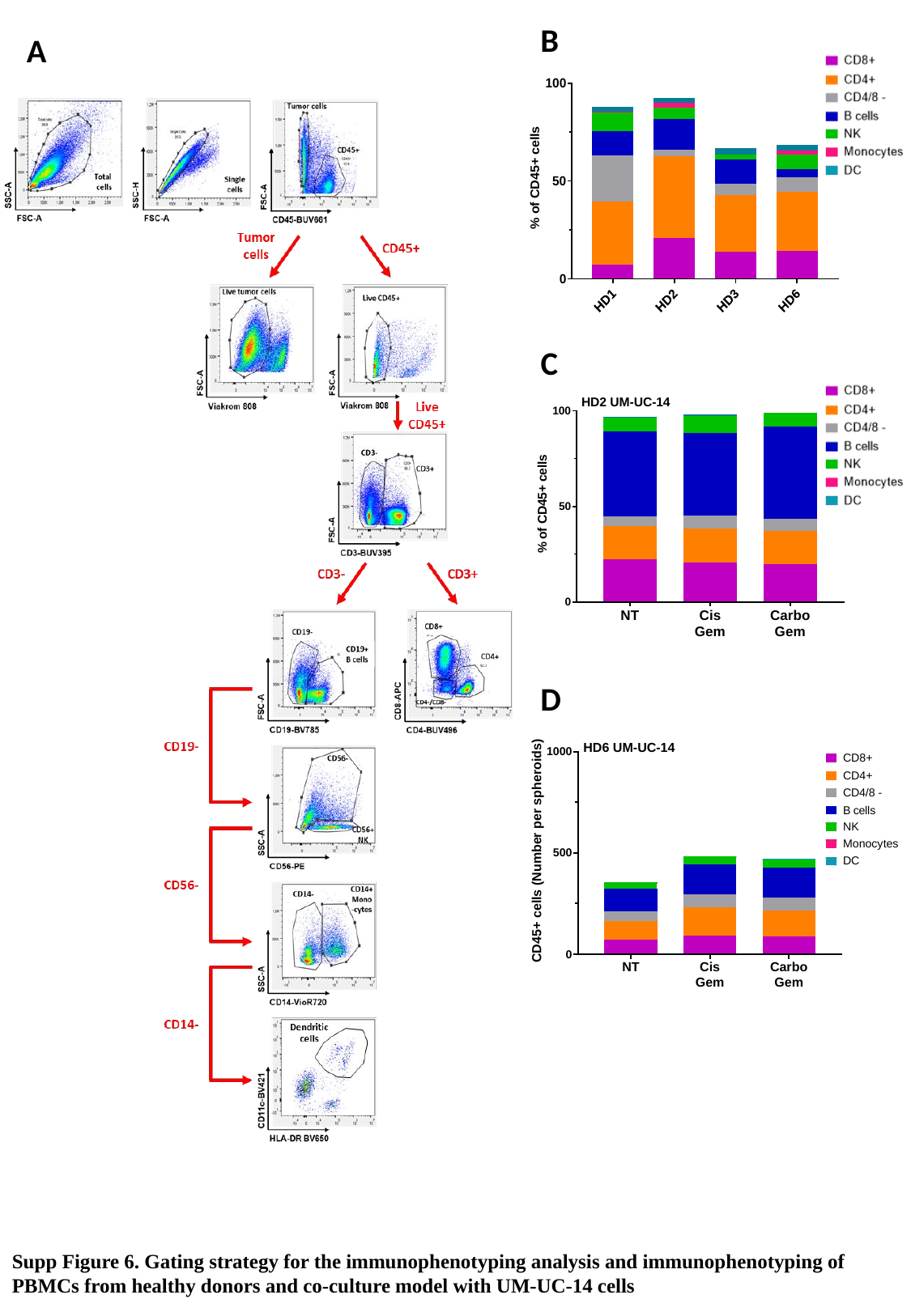

B
A
C
D
Supp Figure 6. Gating strategy for the immunophenotyping analysis and immunophenotyping of PBMCs from healthy donors and co-culture model with UM-UC-14 cells

## Slide 12
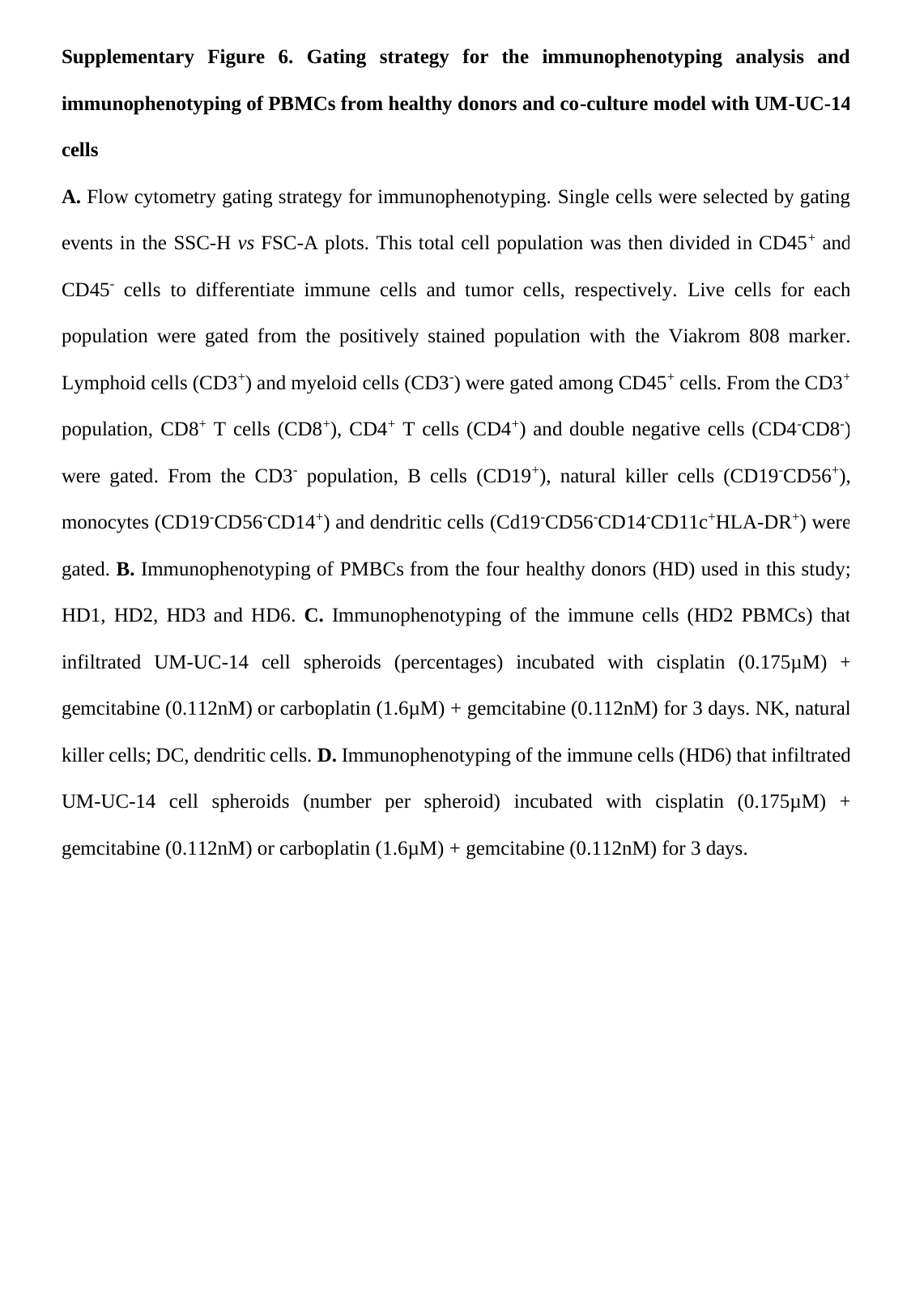

## Slide 13
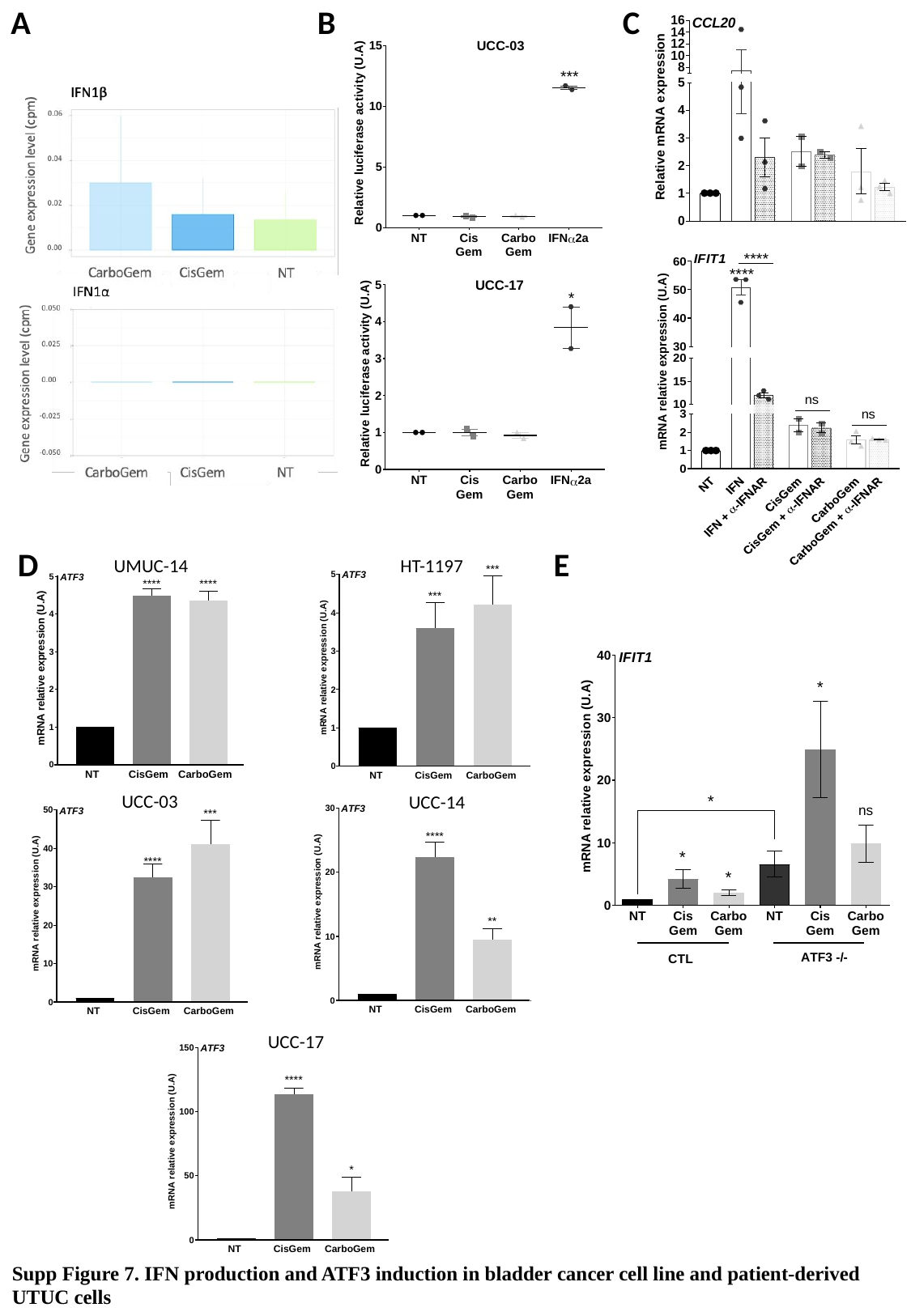

A
B
C
D
E
HT-1197
UMUC-14
UCC-03
UCC-14
UCC-17
Supp Figure 7. IFN production and ATF3 induction in bladder cancer cell line and patient-derived UTUC cells

## Slide 14
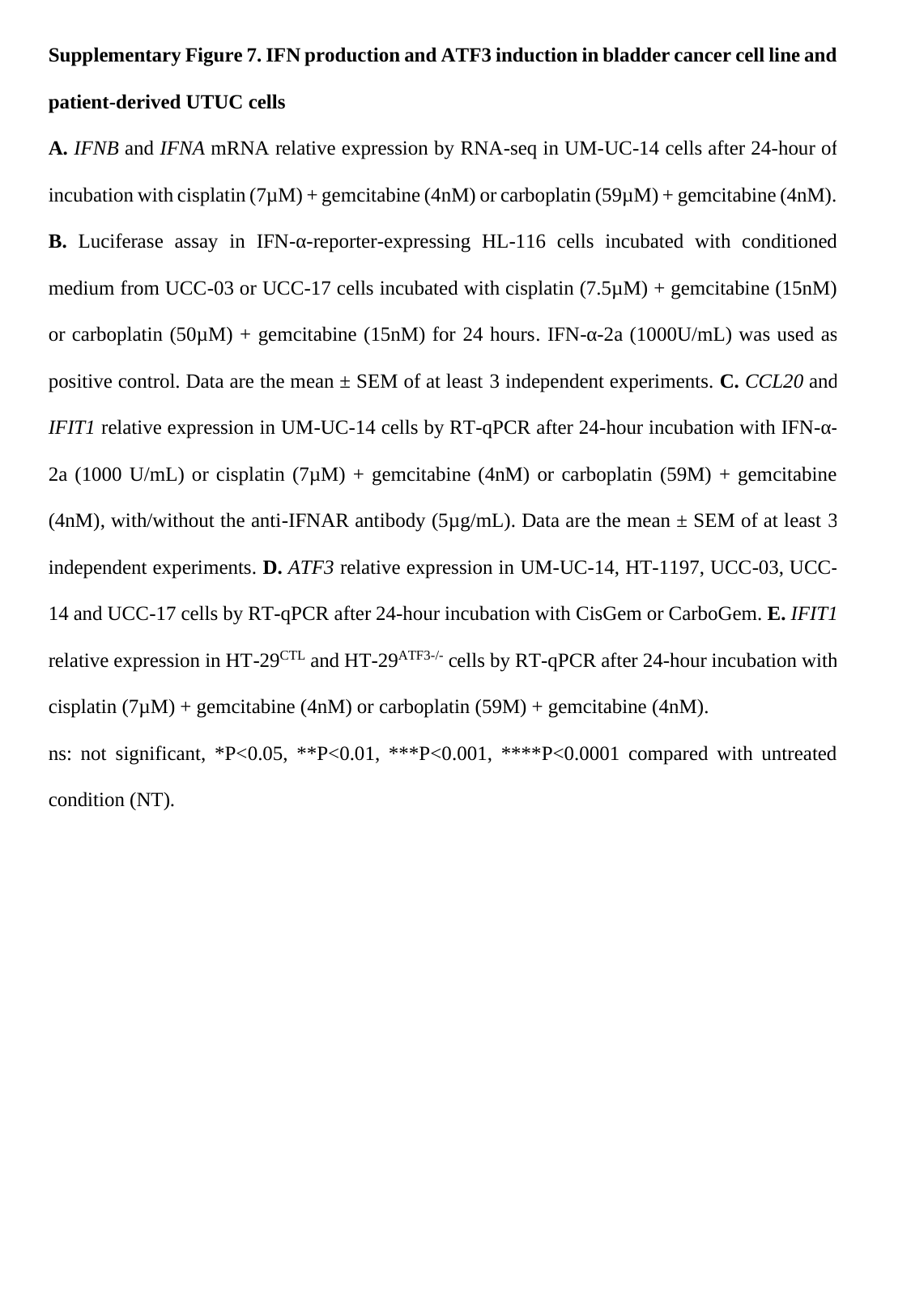

## Slide 15
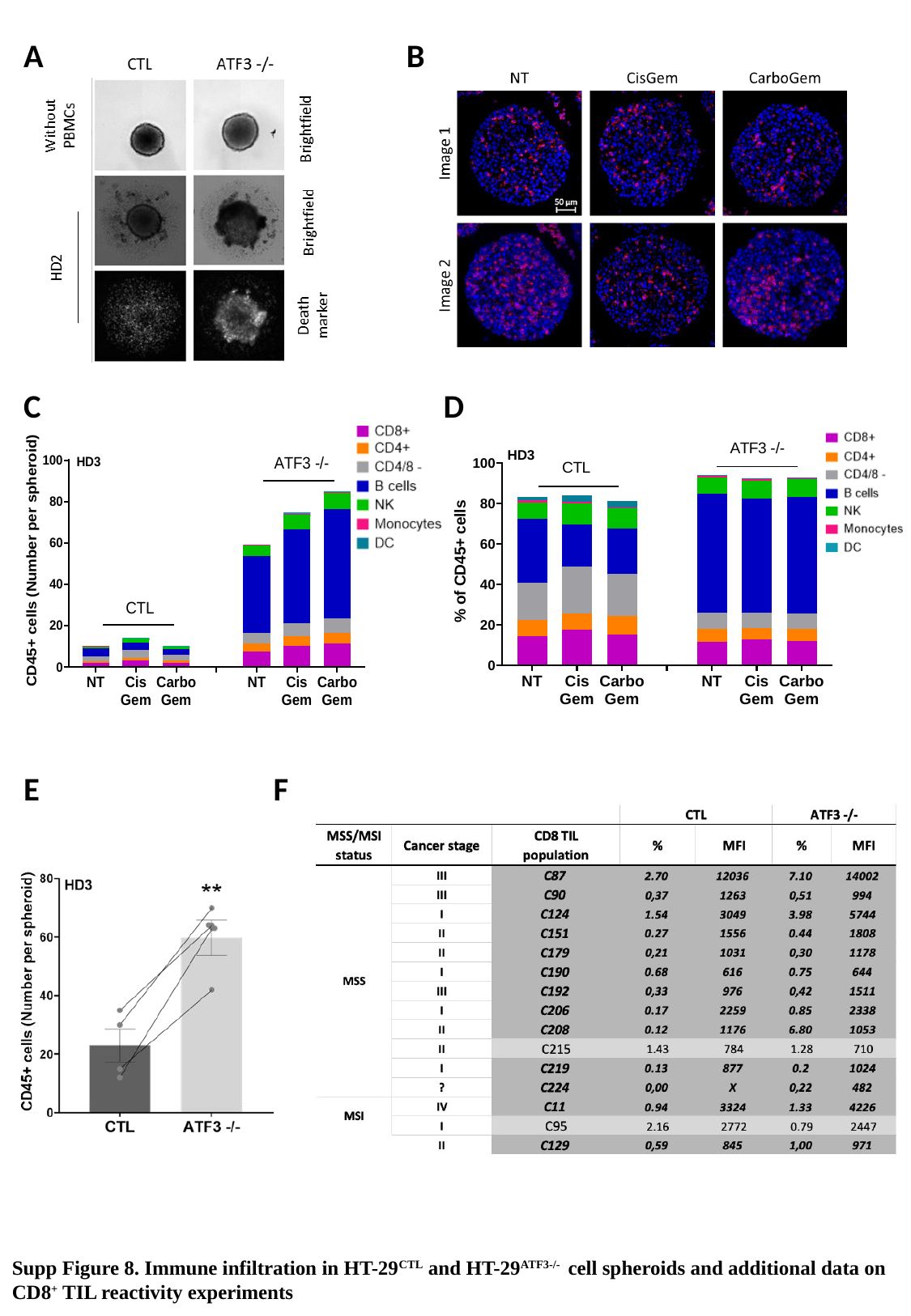

A
B
C
D
E
F
Supp Figure 8. Immune infiltration in HT-29CTL and HT-29ATF3-/- cell spheroids and additional data on CD8+ TIL reactivity experiments

## Slide 16
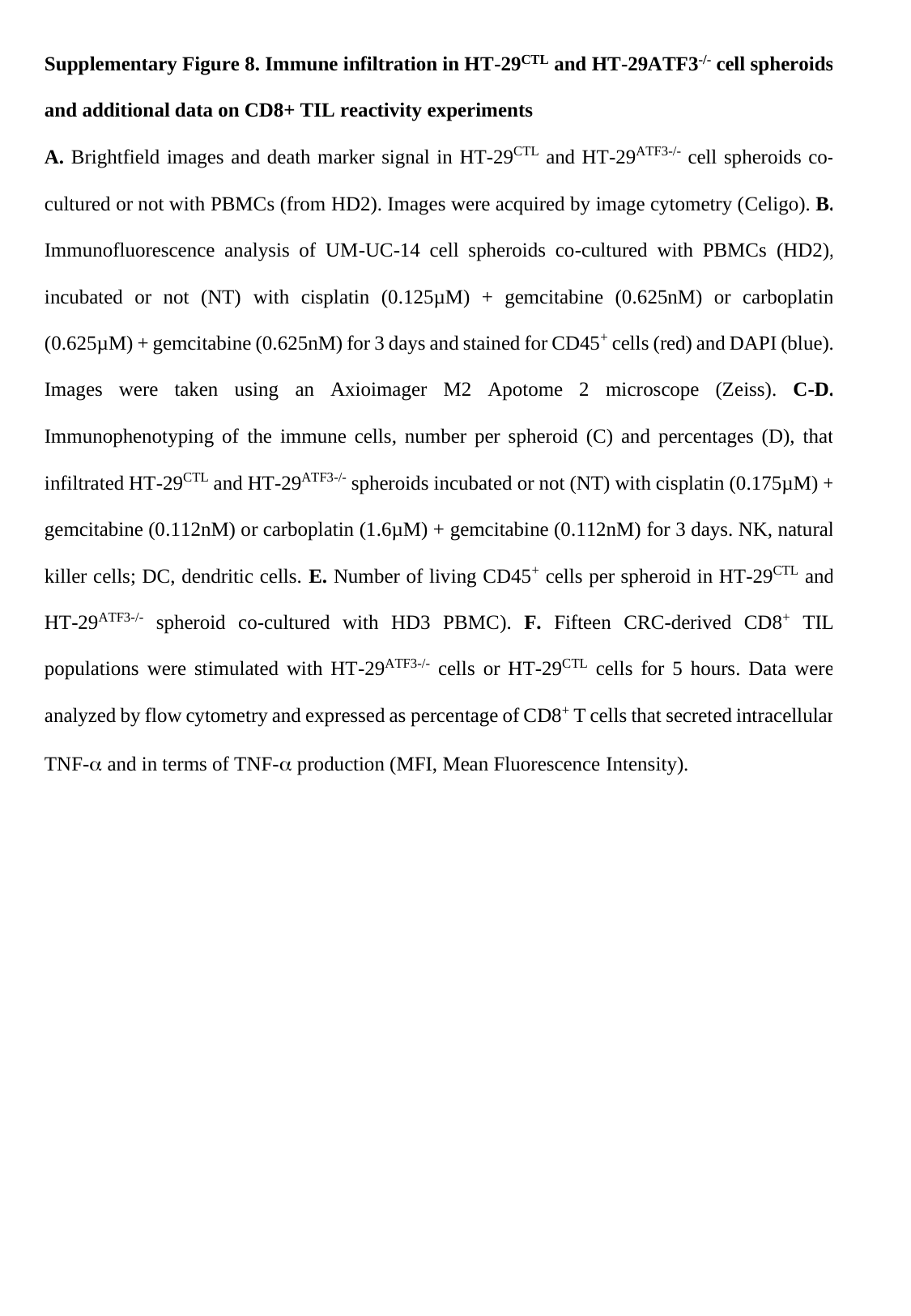

## Slide 17
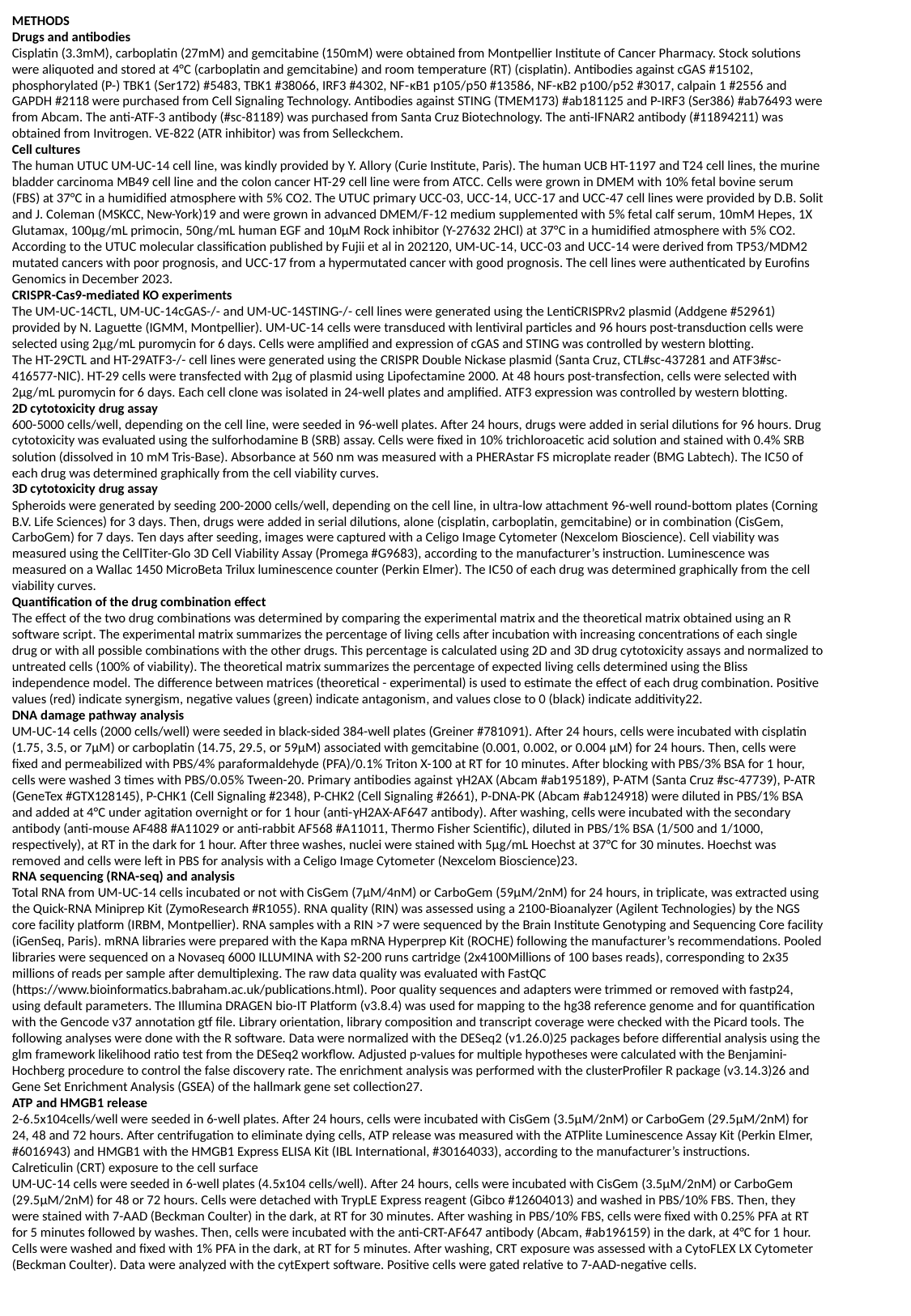

METHODS
Drugs and antibodies
Cisplatin (3.3mM), carboplatin (27mM) and gemcitabine (150mM) were obtained from Montpellier Institute of Cancer Pharmacy. Stock solutions were aliquoted and stored at 4°C (carboplatin and gemcitabine) and room temperature (RT) (cisplatin). Antibodies against cGAS #15102, phosphorylated (P-) TBK1 (Ser172) #5483, TBK1 #38066, IRF3 #4302, NF-κB1 p105/p50 #13586, NF-κB2 p100/p52 #3017, calpain 1 #2556 and GAPDH #2118 were purchased from Cell Signaling Technology. Antibodies against STING (TMEM173) #ab181125 and P-IRF3 (Ser386) #ab76493 were from Abcam. The anti-ATF-3 antibody (#sc-81189) was purchased from Santa Cruz Biotechnology. The anti-IFNAR2 antibody (#11894211) was obtained from Invitrogen. VE-822 (ATR inhibitor) was from Selleckchem.
Cell cultures
The human UTUC UM-UC-14 cell line, was kindly provided by Y. Allory (Curie Institute, Paris). The human UCB HT-1197 and T24 cell lines, the murine bladder carcinoma MB49 cell line and the colon cancer HT-29 cell line were from ATCC. Cells were grown in DMEM with 10% fetal bovine serum (FBS) at 37°C in a humidified atmosphere with 5% CO2. The UTUC primary UCC-03, UCC-14, UCC-17 and UCC-47 cell lines were provided by D.B. Solit and J. Coleman (MSKCC, New-York)19 and were grown in advanced DMEM/F-12 medium supplemented with 5% fetal calf serum, 10mM Hepes, 1X Glutamax, 100µg/mL primocin, 50ng/mL human EGF and 10µM Rock inhibitor (Y-27632 2HCl) at 37°C in a humidified atmosphere with 5% CO2. According to the UTUC molecular classification published by Fujii et al in 202120, UM-UC-14, UCC-03 and UCC-14 were derived from TP53/MDM2 mutated cancers with poor prognosis, and UCC-17 from a hypermutated cancer with good prognosis. The cell lines were authenticated by Eurofins Genomics in December 2023.
CRISPR-Cas9-mediated KO experiments
The UM-UC-14CTL, UM-UC-14cGAS-/- and UM-UC-14STING-/- cell lines were generated using the LentiCRISPRv2 plasmid (Addgene #52961) provided by N. Laguette (IGMM, Montpellier). UM-UC-14 cells were transduced with lentiviral particles and 96 hours post-transduction cells were selected using 2µg/mL puromycin for 6 days. Cells were amplified and expression of cGAS and STING was controlled by western blotting.
The HT-29CTL and HT-29ATF3-/- cell lines were generated using the CRISPR Double Nickase plasmid (Santa Cruz, CTL#sc-437281 and ATF3#sc-416577-NIC). HT-29 cells were transfected with 2µg of plasmid using Lipofectamine 2000. At 48 hours post-transfection, cells were selected with 2µg/mL puromycin for 6 days. Each cell clone was isolated in 24-well plates and amplified. ATF3 expression was controlled by western blotting.
2D cytotoxicity drug assay
600-5000 cells/well, depending on the cell line, were seeded in 96-well plates. After 24 hours, drugs were added in serial dilutions for 96 hours. Drug cytotoxicity was evaluated using the sulforhodamine B (SRB) assay. Cells were fixed in 10% trichloroacetic acid solution and stained with 0.4% SRB solution (dissolved in 10 mM Tris-Base). Absorbance at 560 nm was measured with a PHERAstar FS microplate reader (BMG Labtech). The IC50 of each drug was determined graphically from the cell viability curves.
3D cytotoxicity drug assay
Spheroids were generated by seeding 200-2000 cells/well, depending on the cell line, in ultra-low attachment 96-well round-bottom plates (Corning B.V. Life Sciences) for 3 days. Then, drugs were added in serial dilutions, alone (cisplatin, carboplatin, gemcitabine) or in combination (CisGem, CarboGem) for 7 days. Ten days after seeding, images were captured with a Celigo Image Cytometer (Nexcelom Bioscience). Cell viability was measured using the CellTiter-Glo 3D Cell Viability Assay (Promega #G9683), according to the manufacturer’s instruction. Luminescence was measured on a Wallac 1450 MicroBeta Trilux luminescence counter (Perkin Elmer). The IC50 of each drug was determined graphically from the cell viability curves.
Quantification of the drug combination effect
The effect of the two drug combinations was determined by comparing the experimental matrix and the theoretical matrix obtained using an R software script. The experimental matrix summarizes the percentage of living cells after incubation with increasing concentrations of each single drug or with all possible combinations with the other drugs. This percentage is calculated using 2D and 3D drug cytotoxicity assays and normalized to untreated cells (100% of viability). The theoretical matrix summarizes the percentage of expected living cells determined using the Bliss independence model. The difference between matrices (theoretical - experimental) is used to estimate the effect of each drug combination. Positive values (red) indicate synergism, negative values (green) indicate antagonism, and values close to 0 (black) indicate additivity22.
DNA damage pathway analysis
UM-UC-14 cells (2000 cells/well) were seeded in black-sided 384-well plates (Greiner #781091). After 24 hours, cells were incubated with cisplatin (1.75, 3.5, or 7µM) or carboplatin (14.75, 29.5, or 59µM) associated with gemcitabine (0.001, 0.002, or 0.004 µM) for 24 hours. Then, cells were fixed and permeabilized with PBS/4% paraformaldehyde (PFA)/0.1% Triton X-100 at RT for 10 minutes. After blocking with PBS/3% BSA for 1 hour, cells were washed 3 times with PBS/0.05% Tween-20. Primary antibodies against γH2AX (Abcam #ab195189), P-ATM (Santa Cruz #sc-47739), P-ATR (GeneTex #GTX128145), P-CHK1 (Cell Signaling #2348), P-CHK2 (Cell Signaling #2661), P-DNA-PK (Abcam #ab124918) were diluted in PBS/1% BSA and added at 4°C under agitation overnight or for 1 hour (anti-γH2AX-AF647 antibody). After washing, cells were incubated with the secondary antibody (anti-mouse AF488 #A11029 or anti-rabbit AF568 #A11011, Thermo Fisher Scientific), diluted in PBS/1% BSA (1/500 and 1/1000, respectively), at RT in the dark for 1 hour. After three washes, nuclei were stained with 5µg/mL Hoechst at 37°C for 30 minutes. Hoechst was removed and cells were left in PBS for analysis with a Celigo Image Cytometer (Nexcelom Bioscience)23.
RNA sequencing (RNA-seq) and analysis
Total RNA from UM-UC-14 cells incubated or not with CisGem (7µM/4nM) or CarboGem (59µM/2nM) for 24 hours, in triplicate, was extracted using the Quick-RNA Miniprep Kit (ZymoResearch #R1055). RNA quality (RIN) was assessed using a 2100-Bioanalyzer (Agilent Technologies) by the NGS core facility platform (IRBM, Montpellier). RNA samples with a RIN >7 were sequenced by the Brain Institute Genotyping and Sequencing Core facility (iGenSeq, Paris). mRNA libraries were prepared with the Kapa mRNA Hyperprep Kit (ROCHE) following the manufacturer’s recommendations. Pooled libraries were sequenced on a Novaseq 6000 ILLUMINA with S2-200 runs cartridge (2x4100Millions of 100 bases reads), corresponding to 2x35 millions of reads per sample after demultiplexing. The raw data quality was evaluated with FastQC (https://www.bioinformatics.babraham.ac.uk/publications.html). Poor quality sequences and adapters were trimmed or removed with fastp24, using default parameters. The Illumina DRAGEN bio-IT Platform (v3.8.4) was used for mapping to the hg38 reference genome and for quantification with the Gencode v37 annotation gtf file. Library orientation, library composition and transcript coverage were checked with the Picard tools. The following analyses were done with the R software. Data were normalized with the DESeq2 (v1.26.0)25 packages before differential analysis using the glm framework likelihood ratio test from the DESeq2 workflow. Adjusted p-values for multiple hypotheses were calculated with the Benjamini-Hochberg procedure to control the false discovery rate. The enrichment analysis was performed with the clusterProfiler R package (v3.14.3)26 and Gene Set Enrichment Analysis (GSEA) of the hallmark gene set collection27.
ATP and HMGB1 release
2-6.5x10^4^cells/well were seeded in 6-well plates. After 24 hours, cells were incubated with CisGem (3.5µM/2nM) or CarboGem (29.5µM/2nM) for 24, 48 and 72 hours. After centrifugation to eliminate dying cells, ATP release was measured with the ATPlite Luminescence Assay Kit (Perkin Elmer, #6016943) and HMGB1 with the HMGB1 Express ELISA Kit (IBL International, #30164033), according to the manufacturer’s instructions.
Calreticulin (CRT) exposure to the cell surface
UM-UC-14 cells were seeded in 6-well plates (4.5x10^4^ cells/well). After 24 hours, cells were incubated with CisGem (3.5µM/2nM) or CarboGem (29.5µM/2nM) for 48 or 72 hours. Cells were detached with TrypLE Express reagent (Gibco #12604013) and washed in PBS/10% FBS. Then, they were stained with 7-AAD (Beckman Coulter) in the dark, at RT for 30 minutes. After washing in PBS/10% FBS, cells were fixed with 0.25% PFA at RT for 5 minutes followed by washes. Then, cells were incubated with the anti-CRT-AF647 antibody (Abcam, #ab196159) in the dark, at 4°C for 1 hour. Cells were washed and fixed with 1% PFA in the dark, at RT for 5 minutes. After washing, CRT exposure was assessed with a CytoFLEX LX Cytometer (Beckman Coulter). Data were analyzed with the cytExpert software. Positive cells were gated relative to 7-AAD-negative cells.

## Slide 18
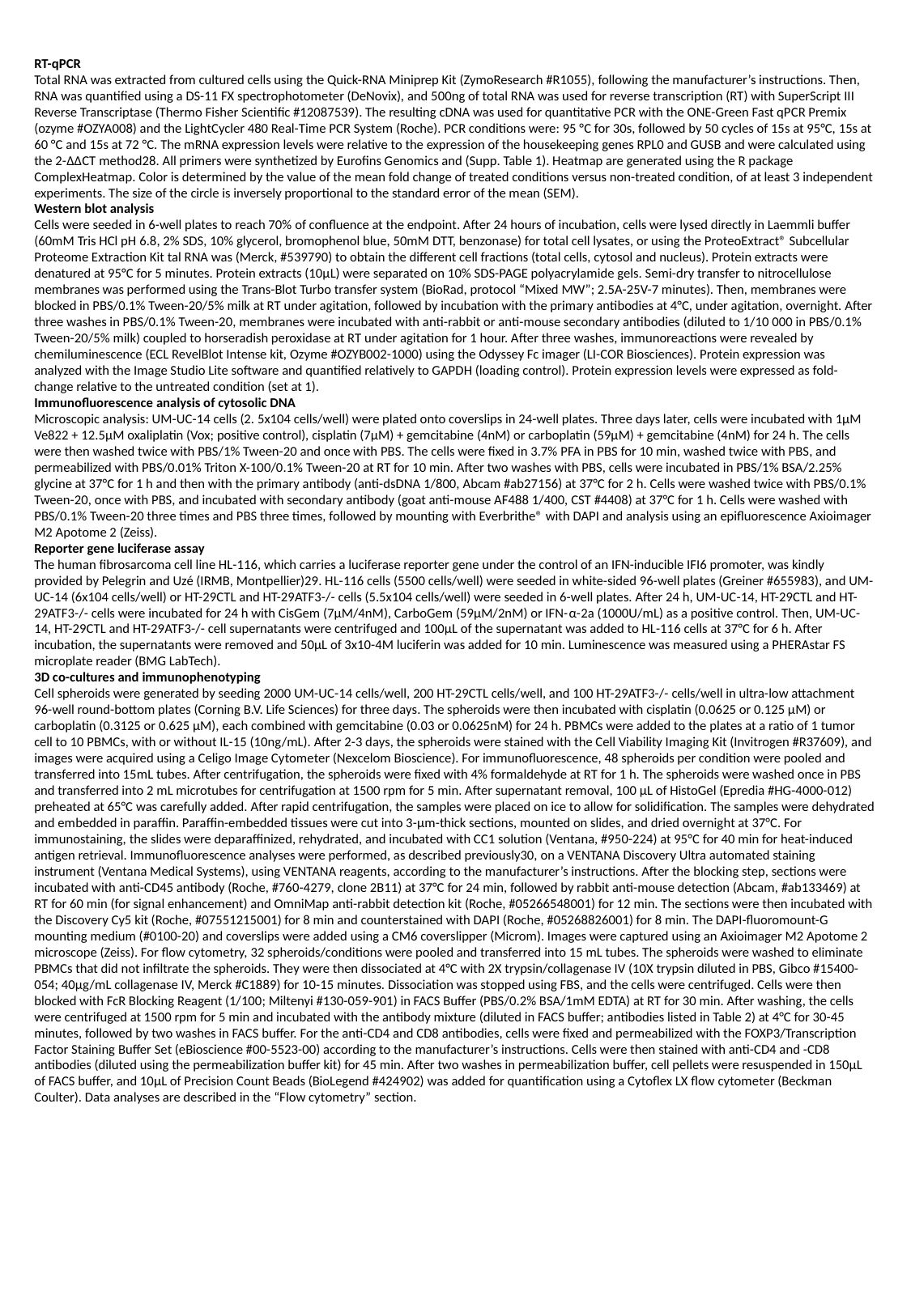

RT-qPCR
Total RNA was extracted from cultured cells using the Quick-RNA Miniprep Kit (ZymoResearch #R1055), following the manufacturer’s instructions. Then, RNA was quantified using a DS-11 FX spectrophotometer (DeNovix), and 500ng of total RNA was used for reverse transcription (RT) with SuperScript III Reverse Transcriptase (Thermo Fisher Scientific #12087539). The resulting cDNA was used for quantitative PCR with the ONE-Green Fast qPCR Premix (ozyme #OZYA008) and the LightCycler 480 Real-Time PCR System (Roche). PCR conditions were: 95 °C for 30s, followed by 50 cycles of 15s at 95°C, 15s at 60 °C and 15s at 72 °C. The mRNA expression levels were relative to the expression of the housekeeping genes RPL0 and GUSB and were calculated using the 2-ΔΔCT method28. All primers were synthetized by Eurofins Genomics and (Supp. Table 1). Heatmap are generated using the R package ComplexHeatmap. Color is determined by the value of the mean fold change of treated conditions versus non-treated condition, of at least 3 independent experiments. The size of the circle is inversely proportional to the standard error of the mean (SEM).
Western blot analysis
Cells were seeded in 6-well plates to reach 70% of confluence at the endpoint. After 24 hours of incubation, cells were lysed directly in Laemmli buffer (60mM Tris HCl pH 6.8, 2% SDS, 10% glycerol, bromophenol blue, 50mM DTT, benzonase) for total cell lysates, or using the ProteoExtract® Subcellular Proteome Extraction Kit tal RNA was (Merck, #539790) to obtain the different cell fractions (total cells, cytosol and nucleus). Protein extracts were denatured at 95°C for 5 minutes. Protein extracts (10µL) were separated on 10% SDS-PAGE polyacrylamide gels. Semi-dry transfer to nitrocellulose membranes was performed using the Trans-Blot Turbo transfer system (BioRad, protocol “Mixed MW”; 2.5A-25V-7 minutes). Then, membranes were blocked in PBS/0.1% Tween-20/5% milk at RT under agitation, followed by incubation with the primary antibodies at 4°C, under agitation, overnight. After three washes in PBS/0.1% Tween-20, membranes were incubated with anti-rabbit or anti-mouse secondary antibodies (diluted to 1/10 000 in PBS/0.1% Tween-20/5% milk) coupled to horseradish peroxidase at RT under agitation for 1 hour. After three washes, immunoreactions were revealed by chemiluminescence (ECL RevelBlot Intense kit, Ozyme #OZYB002-1000) using the Odyssey Fc imager (LI-COR Biosciences). Protein expression was analyzed with the Image Studio Lite software and quantified relatively to GAPDH (loading control). Protein expression levels were expressed as fold-change relative to the untreated condition (set at 1).
Immunofluorescence analysis of cytosolic DNA
Microscopic analysis: UM-UC-14 cells (2. 5x10^4^ cells/well) were plated onto coverslips in 24-well plates. Three days later, cells were incubated with 1µM Ve822 + 12.5µM oxaliplatin (Vox; positive control), cisplatin (7µM) + gemcitabine (4nM) or carboplatin (59µM) + gemcitabine (4nM) for 24 h. The cells were then washed twice with PBS/1% Tween-20 and once with PBS. The cells were fixed in 3.7% PFA in PBS for 10 min, washed twice with PBS, and permeabilized with PBS/0.01% Triton X-100/0.1% Tween-20 at RT for 10 min. After two washes with PBS, cells were incubated in PBS/1% BSA/2.25% glycine at 37°C for 1 h and then with the primary antibody (anti-dsDNA 1/800, Abcam #ab27156) at 37°C for 2 h. Cells were washed twice with PBS/0.1% Tween-20, once with PBS, and incubated with secondary antibody (goat anti-mouse AF488 1/400, CST #4408) at 37°C for 1 h. Cells were washed with PBS/0.1% Tween-20 three times and PBS three times, followed by mounting with Everbrithe® with DAPI and analysis using an epifluorescence Axioimager M2 Apotome 2 (Zeiss).
Reporter gene luciferase assay
The human fibrosarcoma cell line HL-116, which carries a luciferase reporter gene under the control of an IFN-inducible IFI6 promoter, was kindly provided by Pelegrin and Uzé (IRMB, Montpellier)29. HL-116 cells (5500 cells/well) were seeded in white-sided 96-well plates (Greiner #655983), and UM-UC-14 (6x10^4^ cells/well) or HT-29CTL and HT-29ATF3-/- cells (5.5x10^4^ cells/well) were seeded in 6-well plates. After 24 h, UM-UC-14, HT-29CTL and HT-29ATF3-/- cells were incubated for 24 h with CisGem (7µM/4nM), CarboGem (59µM/2nM) or IFN-α-2a (1000U/mL) as a positive control. Then, UM-UC-14, HT-29CTL and HT-29ATF3-/- cell supernatants were centrifuged and 100µL of the supernatant was added to HL-116 cells at 37°C for 6 h. After incubation, the supernatants were removed and 50µL of 3x10^-4^M luciferin was added for 10 min. Luminescence was measured using a PHERAstar FS microplate reader (BMG LabTech).
3D co-cultures and immunophenotyping
Cell spheroids were generated by seeding 2000 UM-UC-14 cells/well, 200 HT-29CTL cells/well, and 100 HT-29ATF3-/- cells/well in ultra-low attachment 96-well round-bottom plates (Corning B.V. Life Sciences) for three days. The spheroids were then incubated with cisplatin (0.0625 or 0.125 µM) or carboplatin (0.3125 or 0.625 µM), each combined with gemcitabine (0.03 or 0.0625nM) for 24 h. PBMCs were added to the plates at a ratio of 1 tumor cell to 10 PBMCs, with or without IL-15 (10ng/mL). After 2-3 days, the spheroids were stained with the Cell Viability Imaging Kit (Invitrogen #R37609), and images were acquired using a Celigo Image Cytometer (Nexcelom Bioscience). For immunofluorescence, 48 spheroids per condition were pooled and transferred into 15mL tubes. After centrifugation, the spheroids were fixed with 4% formaldehyde at RT for 1 h. The spheroids were washed once in PBS and transferred into 2 mL microtubes for centrifugation at 1500 rpm for 5 min. After supernatant removal, 100 µL of HistoGel (Epredia #HG-4000-012) preheated at 65°C was carefully added. After rapid centrifugation, the samples were placed on ice to allow for solidification. The samples were dehydrated and embedded in paraffin. Paraffin-embedded tissues were cut into 3-µm-thick sections, mounted on slides, and dried overnight at 37°C. For immunostaining, the slides were deparaffinized, rehydrated, and incubated with CC1 solution (Ventana, #950-224) at 95°C for 40 min for heat-induced antigen retrieval. Immunofluorescence analyses were performed, as described previously30, on a VENTANA Discovery Ultra automated staining instrument (Ventana Medical Systems), using VENTANA reagents, according to the manufacturer’s instructions. After the blocking step, sections were incubated with anti-CD45 antibody (Roche, #760-4279, clone 2B11) at 37°C for 24 min, followed by rabbit anti-mouse detection (Abcam, #ab133469) at RT for 60 min (for signal enhancement) and OmniMap anti-rabbit detection kit (Roche, #05266548001) for 12 min. The sections were then incubated with the Discovery Cy5 kit (Roche, #07551215001) for 8 min and counterstained with DAPI (Roche, #05268826001) for 8 min. The DAPI-fluoromount-G mounting medium (#0100-20) and coverslips were added using a CM6 coverslipper (Microm). Images were captured using an Axioimager M2 Apotome 2 microscope (Zeiss). For flow cytometry, 32 spheroids/conditions were pooled and transferred into 15 mL tubes. The spheroids were washed to eliminate PBMCs that did not infiltrate the spheroids. They were then dissociated at 4°C with 2X trypsin/collagenase IV (10X trypsin diluted in PBS, Gibco #15400-054; 40µg/mL collagenase IV, Merck #C1889) for 10-15 minutes. Dissociation was stopped using FBS, and the cells were centrifuged. Cells were then blocked with FcR Blocking Reagent (1/100; Miltenyi #130-059-901) in FACS Buffer (PBS/0.2% BSA/1mM EDTA) at RT for 30 min. After washing, the cells were centrifuged at 1500 rpm for 5 min and incubated with the antibody mixture (diluted in FACS buffer; antibodies listed in Table 2) at 4°C for 30-45 minutes, followed by two washes in FACS buffer. For the anti-CD4 and CD8 antibodies, cells were fixed and permeabilized with the FOXP3/Transcription Factor Staining Buffer Set (eBioscience #00-5523-00) according to the manufacturer’s instructions. Cells were then stained with anti-CD4 and -CD8 antibodies (diluted using the permeabilization buffer kit) for 45 min. After two washes in permeabilization buffer, cell pellets were resuspended in 150µL of FACS buffer, and 10µL of Precision Count Beads (BioLegend #424902) was added for quantification using a Cytoflex LX flow cytometer (Beckman Coulter). Data analyses are described in the “Flow cytometry” section.

## Slide 19
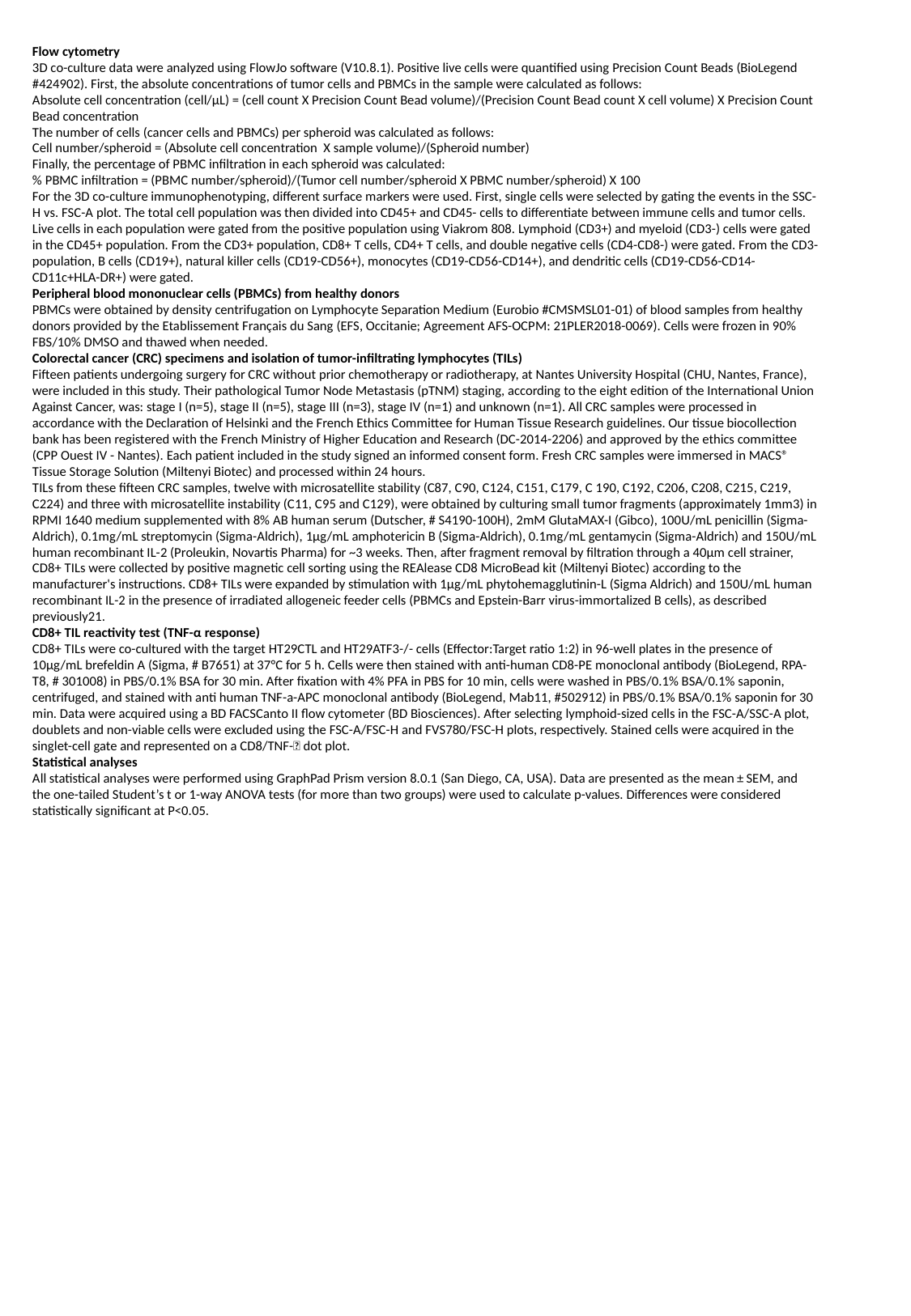

Flow cytometry
3D co-culture data were analyzed using FlowJo software (V10.8.1). Positive live cells were quantified using Precision Count Beads (BioLegend #424902). First, the absolute concentrations of tumor cells and PBMCs in the sample were calculated as follows:
Absolute cell concentration (cell/µL) = (cell count X Precision Count Bead volume)/(Precision Count Bead count X cell volume) X Precision Count Bead concentration
The number of cells (cancer cells and PBMCs) per spheroid was calculated as follows:
Cell number/spheroid = (Absolute cell concentration X sample volume)/(Spheroid number)
Finally, the percentage of PBMC infiltration in each spheroid was calculated:
% PBMC infiltration = (PBMC number/spheroid)/(Tumor cell number/spheroid X PBMC number/spheroid) X 100
For the 3D co-culture immunophenotyping, different surface markers were used. First, single cells were selected by gating the events in the SSC-H vs. FSC-A plot. The total cell population was then divided into CD45+ and CD45- cells to differentiate between immune cells and tumor cells. Live cells in each population were gated from the positive population using Viakrom 808. Lymphoid (CD3+) and myeloid (CD3-) cells were gated in the CD45+ population. From the CD3+ population, CD8+ T cells, CD4+ T cells, and double negative cells (CD4-CD8-) were gated. From the CD3- population, B cells (CD19+), natural killer cells (CD19-CD56+), monocytes (CD19-CD56-CD14+), and dendritic cells (CD19-CD56-CD14-CD11c+HLA-DR+) were gated.
Peripheral blood mononuclear cells (PBMCs) from healthy donors
PBMCs were obtained by density centrifugation on Lymphocyte Separation Medium (Eurobio #CMSMSL01-01) of blood samples from healthy donors provided by the Etablissement Français du Sang (EFS, Occitanie; Agreement AFS-OCPM: 21PLER2018-0069). Cells were frozen in 90% FBS/10% DMSO and thawed when needed.
Colorectal cancer (CRC) specimens and isolation of tumor-infiltrating lymphocytes (TILs)
Fifteen patients undergoing surgery for CRC without prior chemotherapy or radiotherapy, at Nantes University Hospital (CHU, Nantes, France), were included in this study. Their pathological Tumor Node Metastasis (pTNM) staging, according to the eight edition of the International Union Against Cancer, was: stage I (n=5), stage II (n=5), stage III (n=3), stage IV (n=1) and unknown (n=1). All CRC samples were processed in accordance with the Declaration of Helsinki and the French Ethics Committee for Human Tissue Research guidelines. Our tissue biocollection bank has been registered with the French Ministry of Higher Education and Research (DC-2014-2206) and approved by the ethics committee (CPP Ouest IV - Nantes). Each patient included in the study signed an informed consent form. Fresh CRC samples were immersed in MACS® Tissue Storage Solution (Miltenyi Biotec) and processed within 24 hours.
TILs from these fifteen CRC samples, twelve with microsatellite stability (C87, C90, C124, C151, C179, C 190, C192, C206, C208, C215, C219, C224) and three with microsatellite instability (C11, C95 and C129), were obtained by culturing small tumor fragments (approximately 1mm3) in RPMI 1640 medium supplemented with 8% AB human serum (Dutscher, # S4190-100H), 2mM GlutaMAX-I (Gibco), 100U/mL penicillin (Sigma-Aldrich), 0.1mg/mL streptomycin (Sigma-Aldrich), 1µg/mL amphotericin B (Sigma-Aldrich), 0.1mg/mL gentamycin (Sigma-Aldrich) and 150U/mL human recombinant IL-2 (Proleukin, Novartis Pharma) for ~3 weeks. Then, after fragment removal by filtration through a 40µm cell strainer, CD8+ TILs were collected by positive magnetic cell sorting using the REAlease CD8 MicroBead kit (Miltenyi Biotec) according to the manufacturer's instructions. CD8+ TILs were expanded by stimulation with 1µg/mL phytohemagglutinin-L (Sigma Aldrich) and 150U/mL human recombinant IL-2 in the presence of irradiated allogeneic feeder cells (PBMCs and Epstein-Barr virus-immortalized B cells), as described previously21.
CD8+ TIL reactivity test (TNF-α response)
CD8+ TILs were co-cultured with the target HT29CTL and HT29ATF3-/- cells (Effector:Target ratio 1:2) in 96-well plates in the presence of 10µg/mL brefeldin A (Sigma, # B7651) at 37°C for 5 h. Cells were then stained with anti-human CD8-PE monoclonal antibody (BioLegend, RPA-T8, # 301008) in PBS/0.1% BSA for 30 min. After fixation with 4% PFA in PBS for 10 min, cells were washed in PBS/0.1% BSA/0.1% saponin, centrifuged, and stained with anti human TNF-a-APC monoclonal antibody (BioLegend, Mab11, #502912) in PBS/0.1% BSA/0.1% saponin for 30 min. Data were acquired using a BD FACSCanto II flow cytometer (BD Biosciences). After selecting lymphoid-sized cells in the FSC-A/SSC-A plot, doublets and non-viable cells were excluded using the FSC-A/FSC-H and FVS780/FSC-H plots, respectively. Stained cells were acquired in the singlet-cell gate and represented on a CD8/TNF-α dot plot.
Statistical analyses
All statistical analyses were performed using GraphPad Prism version 8.0.1 (San Diego, CA, USA). Data are presented as the mean ± SEM, and the one-tailed Student’s t or 1-way ANOVA tests (for more than two groups) were used to calculate p-values. Differences were considered statistically significant at P<0.05.
